# Identifying and mathematically modeling the time-course of extracellular metabolic markers associated with resistance to ceftolozane/tazobactam in *Pseudomonas aeruginosa*

**DOI:** 10.1128/aac.01081-23

**Published:** 2024-02-20

**Authors:** Jessica R. Tait, Dovile Anderson, Roger L. Nation, Darren J. Creek, Cornelia B. Landersdorfer

**Affiliations:** 1 Drug Delivery, Disposition and Dynamics, Monash Institute of Pharmaceutical Sciences, Monash University, Parkville, Victoria, Australia; Providence Portland Med Ctr, Portland, Oregon, USA

**Keywords:** metabolomics, antibiotic resistance, *Pseudomonas aeruginosa*, mathematical modeling, dynamic *in vitro* model

## Abstract

Extracellular bacterial metabolites have potential as markers of bacterial growth and resistance emergence but have not been evaluated in dynamic *in vitro* studies. We investigated the dynamic metabolomic footprint of a multidrug-resistant hypermutable *Pseudomonas aeruginosa* isolate exposed to ceftolozane/tazobactam as continuous infusion (4.5 g/day, 9 g/day) in a hollow-fiber infection model over 7–9 days in biological replicates (*n* = 5). Bacterial samples were collected at 0, 7, 23, 47, 71, 95, 143, 167, 191, and 215 h, the supernatant quenched, and extracellular metabolites extracted. Metabolites were analyzed via untargeted metabolomics, including hierarchical clustering and correlation with quantified total and resistant bacterial populations. The time-courses of five (of 1,921 detected) metabolites from enriched pathways were mathematically modeled. Absorbed L-arginine and secreted L-ornithine were highly correlated with the total bacterial population (r −0.79 and 0.82, respectively, *P*<0.0001). Ribose-5-phosphate, sedoheptulose-7-phosphate, and trehalose-6-phosphate correlated with the resistant subpopulation (0.64, 0.64, and 0.67, respectively, *P*<0.0001) and were likely secreted due to resistant growth overcoming oxidative and osmotic stress induced by ceftolozane/tazobactam. Using pharmacokinetic/pharmacodynamic-based transduction models, these metabolites were successfully modeled based on the total or resistant bacterial populations. The models well described the abundance of each metabolite across the differing time-course profiles of biological replicates, based on bacterial killing and, importantly, resistant regrowth. These proof-of-concept studies suggest that further exploration is warranted to determine the generalizability of these findings. The metabolites modeled here are not exclusive to bacteria. Future studies may use this approach to identify bacteria-specific metabolites correlating with resistance, which would ultimately be extremely useful for clinical translation.

## INTRODUCTION

Antibiotic resistance is a serious threat to global health and a particular burden for vulnerable patient populations such as those with cystic fibrosis (CF) or the critically ill (1). The optimization of antibiotic dosing regimens has mostly relied on traditional pharmacokinetic/pharmacodynamic (PK/PD) indices. The index of an antibiotic relies on the PK exposure (i.e., a time-collapsed summary measure of the antibiotic concentrations in a patient) and the PD measure of the minimum inhibitory concentration (MIC) (i.e., the antibiotic concentration required to inhibit visible growth of the infecting bacteria) (2). Therapeutic drug monitoring (TDM) is employed routinely for certain antibiotics and in select high-risk cases for others (3). It involves measuring antibiotic concentrations in the blood of a patient, ideally with multiple samples throughout a dosing interval (4). Dosing can be adjusted based on the TDM results and the target exposure associated with the relevant PK/PD index. The MICs of clinical isolates are often determined for serious infections at onset but have well-recognized limitations such as high inter- and intra-laboratory variability and inability to describe bacterial characteristics like hypermutability and heteroresistance (5–8). Beyond pathogen identification and susceptibility testing, molecular rapid diagnostic tests can be employed at onset of infection to identify the presence of resistance mechanisms and assist in the decision of which antibiotics to use for treatment (9, 10). Procalcitonin, a host-based biomarker, can assist clinicians in deciding when to start and discontinue antibacterial treatment for patients with sepsis but has its own limitations (11). However, there is a lack of bacterial-based markers to monitor the progression of bacterial killing and emergence of resistance over the course of infection and treatment.

Metabolomics is a rapidly advancing field in clinical and laboratory microbiology (12). Major areas of metabolomics research with *Pseudomonas aeruginosa* include identifying and differentiating bacterial strains, associating metabolic traits with genome structure, and identifying new metabolites and metabolic changes in response to external factors (e.g., growth conditions or antibiotic challenge) (13). Perturbations in the metabolome are downstream from genomic, transcriptomic, and proteomic shifts and are most indicative of a phenotypic response to environmental changes (14). Extracellular metabolomics (also referred to as metabolic footprinting or exometabolomics) provide insight into the uptake and secretion of metabolites for the pathogen in question, which are subject to vast changes depending on the environment (15). An extracellular metabolite could hypothetically be used as a biomarker, specifically, a monitoring marker that assesses the impact of antibiotic therapy on the bacterial population when repeatedly measured (16).

We hypothesized that extracellular metabolic markers could be used to quantitatively monitor the bacterial population, including bacterial killing and resistance emergence following antibiotic exposure, and, to this end, conducted *in vitro* PK/PD infection model studies. We challenged a hypermutable multidrug-resistant (MDR) *P. aeruginosa* clinical isolate with ceftolozane-tazobactam via continuous infusion over 7–9 days in the hollow-fiber infection model (HFIM), as recently published (8). From those studies, we collected samples for extracellular metabolomics and performed dynamic metabolomic footprint analysis over the treatment period to identify potential monitoring markers to measure the bacterial response to antibiotic exposure. We also used mathematical modeling to describe the relationships between the time-courses of bacterial response [modeled previously (8)] and the metabolic footprint of each of five metabolites.

## MATERIALS AND METHODS

### Hollow-fiber infection model

A hypermutable *P. aeruginosa* clinical isolate, CW41, was challenged with ceftolozane-tazobactam (Zerbaxa, MSD, Australia) in the HFIM (C3008-1 cartridges; FiberCell Systems Inc., Frederick, MD, USA), in five biological replicates performed across two studies. The first study, with replicates 1 and 2, was conducted over 167 h and the second study, with replicates 3, 4, and 5, over 215 h. The setup of the HFIM is represented in Fig. S1. The methods and bacterial results of these HFIM studies are described in detail in a previous publication (8). Briefly, the studied isolate was characterized as susceptible to ceftolozane-tazobactam (MIC 4 mg/L), and MDR (i.e., resistant to at least one antibiotic from each of ≥3 antibiotic classes) (17–20). The HFIM studies used cation-adjusted Mueller Hinton broth (CAMHB) and agar (CAMHA) [Becton Dickinson & Co., Sparks, MD, USA, with 25.0 mg/L of Ca^2+^ and 12.5 mg/L of Mg^2+^]. Ceftolozane-tazobactam was administered to simulate steady-state concentrations of ceftolozane predicted to occur in the epithelial lining fluid of the lung in patients with CF, following daily doses of 3 g/1.5 g and 6 g/3 g via continuous infusion (10.6 and 21.3 mg/L, respectively) (21–23). Total bacterial populations were quantified on antibiotic-free CAMHA and resistant subpopulations on CAMHA containing ceftolozane-tazobactam (12 and 20 mg/L).

For metabolomic footprint analysis, the supernatant from centrifuged bacterial samples was collected. These bacterial samples were obtained from the hollow-fiber cartridges for all untreated control and treated arms for each of the five biological replicates at 7, 23, 47, 71, 143, and 167 h. Additional bacterial samples were collected from the cartridges at 0 and 215 h for replicates 3 to 5. Matrix blank samples (of CAMHB not containing any bacteria) were collected from the diluent media bottles every 48 h for all untreated control and treated arms.

### Preparations of extracellular metabolite extracts

The bacterial supernatant samples and matrix blank samples were processed for metabolomic footprint analysis (24). See the supplementary materials for diagrams of the sample processing (Fig. S2A) and data acquisition and processing (Fig. S2B). Briefly, each sample (25 µL) was added to 100 µL of pre-chilled methanol containing the internal standards (CHAPS, CAPS, TRIS, and PIPES) at 1 µM. This mixture was vortexed and subsequently centrifuged at 14,800× *g* and 4°C for 10 min (Fig. S2A). The final supernatant samples containing the extracted extracellular metabolites were stored at −80°C until liquid chromatography-mass spectrometry (LC-MS) analysis was performed (Fig. S2B).

### LC-MS analysis

LC-MS data were acquired on a Q-Exactive Orbitrap mass spectrometer (Thermo Fisher) coupled with a high-performance liquid chromatography (HPLC) system Dionex Ultimate 3000 RS (Thermo Fisher). Chromatographic separation was performed on a ZIC-pHILIC column (5 µm, polymeric, 150 × 4.6 mm, SeQuant, Merck). The mobile phases were (A) 20 mM of ammonium carbonate and (B) acetonitrile. The gradient program started at 80% B, which was reduced to 50% B over 15 min, then reduced from 50% B to 5% B over 3 min, followed by wash with 5% B for another 3 min, and finally an 8-min re-equilibration with 80% B. The flow rate was 0.3 mL/min, and the column compartment temperature was 25°C. The total run time was 32 min with an injection sample volume of 10 µL. The mass spectrometer operated in full scan mode with positive and negative polarity switching at 35,000 resolution at 200 *m/z* with a detection range of 85 to 1,275 *m/z* in full scan mode. The electrospray ionization source (HESI) was set to 3.5 kV voltage for positive mode and 4.0 kV for negative mode, sheath gas was set to 50 and auxiliary gas to 20 arbitrary units, capillary temperature was 300°C, and probe heater temperature was 120°C.

The samples were analyzed in two LC-MS runs, the first for replicates 1 and 2 and the second for replicates 3, 4, and 5 (Fig. S2B). Samples were analyzed in randomized order and pooled quality control (PQC) samples were periodically analyzed throughout each LC-MS run to assess reproducibility. PQC samples were pooled mixtures of aliquots from all samples of study 1 and another mixture of aliquots from study 2 samples. Mixtures of pure authentic standards containing over 400 metabolites were analyzed as separate injections and used to confirm retention times. Metabolites confirmed with reference standards were given confidence MSI level 1 (25), while others were putatively annotated based on accurate mass and predicted retention time (MSI Level 2) (26).

### Metabolomic data processing

See Fig. S2B for an overview diagram of the data acquisition and processing. The acquired LC-MS data were processed in untargeted fashion using an open-source software IDEOM (http://mzmatch.sourceforge.net/ideom.php) (27), which initially used *ProteoWizard* to convert raw LC-MS files to *mzXML* format and *XCMS* to pick peaks (28). *Mzmatch.R* was subsequently used to convert to *peakML* files, align samples and filter peaks using minimum detectable peak height intensity of 100,000, relative standard deviation (RSD) of <0.5 (reproducibility), and peak shape (codadw) of >0.8 (29). Peaks with intensity below the minimum peak height intensity of 100,000 were fixed to that threshold. *Mzmatch* was also used to retrieve missing peaks and for annotation of related peaks. Default IDEOM parameters were used to eliminate unwanted noise and artefact peaks. Loss or gain of a proton was corrected in negative and positive ESI mode, respectively, followed by putative identification of metabolites by accurate mass within 3 ppm mass error searching against the Human Metabolome Database (HMDB), Kyoto Encyclopedia of Genes and Genomes (KEGG), MetaCyc, and LIPIDMAPS databases (30–33).

Relative abundance was calculated as the measured peak height intensity divided by the average peak height intensity for each metabolite per LC-MS run. Data from both runs (i.e., study 1 and study 2) were combined, and unsupervised multivariate analysis was performed. For multivariate analysis, relative abundance data were log-transformed and auto-scaled to achieve normal distribution of the data. Principal component analysis (PCA) and hierarchical clustering analysis (HCA) were performed in Metaboanalyst 5.0 (34). Pearson correlation analysis was performed between the relative abundance of extracellular metabolites and bacterial pharmacodynamic metrics including total population (log_10_CFU/mL on antibiotic-free CAMHA), resistant subpopulation [log_10_CFU/mL on CAMHA containing ceftolozane-tazobactam (20 mg/L)], and log_10_-change (of the total population at time *t*; log_10_CFU/mL*_t_* – log_10_CFU/mL_0h_). Metabolites identified by correlation analysis were subjected to further manual filtering, which considered the magnitude of relative abundance and pathway enrichment analysis with KEGG pathways (31), to identify candidates for mathematical modeling. Extracellular ornithine, arginine, D-ribose 5-phosphate, D-sedoheptulose 7-phosphate, and trehalose 6-phosphate, which were among the metabolites confirmed with reference standards, were selected for mathematical modeling.

### Mathematical modeling

Mathematical modeling of the selected extracellular metabolites measured in the HFIM was performed using S-ADAPT software with SADAPT-TRAN (35, 36). Biological replicates were recorded separately in the metabolomic data set and simultaneously modeled. Metabolite models were initially developed using replicates 1 and 2 from the first study, while the final modeling was performed with all data (i.e., five replicates). The metabolomic data were fitted by an additive residual error model. Models were evaluated based on goodness-of-fit, the S-ADAPT objective function value (−1∙log likelihood), Akaike information criterion (AIC; −2∙log likelihood +2∙number of parameters), and standard diagnostic plots. The relative bias and relative root mean squared error were calculated (37, 38).

**Fig 1:**
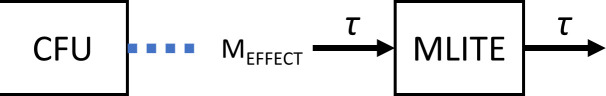
Schematic of the transduction model structure used to translate between bacterial data (CFU; either CFUALL or CFUonDP20) and metabolomic data (MLITE) in the HFIM, via a theoretical effect (MEFFECT) driven by the bacterial compartment (CFU) that transited (τ) to the metabolite compartment (MLITE).

Biophase and transduction PK/PD models (Fig. S3; Fig. 1, respectively) were considered during model development to link the bacterial model described previously (8) to changes in relative abundance of the extracellular metabolites quantified in the HFIM. Based on the evaluation criteria above, a transduction model structure was used (Fig. 1). Transduction models were developed using either the total population of bacteria on antibiotic-free CAMHA [log_10_ (CFU_ALL_)] or the resistant subpopulation on CAMHA containing ceftolozane-tazobactam [20 mg/L, log_10_ (CFUonDP_20_)] from the previous model (8) as the input to the sigmoidal effect model (*M*_EFFECT_) for the metabolites ornithine, D-ribose 5-phosphate, D-sedoheptulose 7-phosphate, and trehalose 6-phosphate:


$$M_{EFFECT}\;=\;\;\frac{E_{MAX}\;\cdot \;log_{10}{\left(CFU\right)}^{HILL}}{log_{10}{\left(CFU\right)}^{HILL}\;+\;EC_{50}^{HILL}}$$

where CFU was CFU_ALL_ for ornithine and was CFUonDP_20_ for D-ribose 5-phosphate, trehalose 6-phosphate, and D-sedoheptulose 7-phosphate.

For arginine, an inhibitory effect with a baseline parameter (*E*_0_) was used:


$$M_{EFFECT}\;=\;E_{0}-\;\;\frac{E_{MAX}\;\cdot \;log_{10}{\left(CFU_{ALL}\right)}^{HILL}}{log_{10}{\left(CFU_{ALL}\right)}^{HILL}\;+\;EC_{50}^{HILL}}$$

The metabolite concentrations (MLITE, quantified as relative abundance) were described by the following differential equation:


$$\frac{d\left(MLITE\right)}{dt}=\left(M_{EFFECT}-MLITE\right)/\tau$$

where τ was the transition time (h) to and from the metabolite compartment.

## RESULTS

### Extracellular metabolites

A total of 91 extracellular metabolites were annotated in IDEOM software, which included 37 with MSI level 1 identification. An additional 1,830 putative metabolites were detected but were excluded from further analysis due to detection in only one study, low confidence annotation, or annotation as short peptides or other metabolites that were not mapped to KEGG metabolic pathways. Data precision was confirmed by the median RSD of bacterial supernatant and broth samples, and PQC samples (Table S1), as well as clustering of PQC samples in the PCA plot (Fig. S4). The PCA plots exhibited separation between the control and treatments at earlier timepoints, which was retained for the 6 g/3 g treatment until 95 h (Fig. S4). HCA was more descriptive of the dynamic profiles of the 91 extracellular metabolites compared to PCA. Specifically, the heatmap with HCA revealed clusters of extracellular metabolites with distinct trends over the time-course of the studies (Fig. S5).

**Fig 2:**
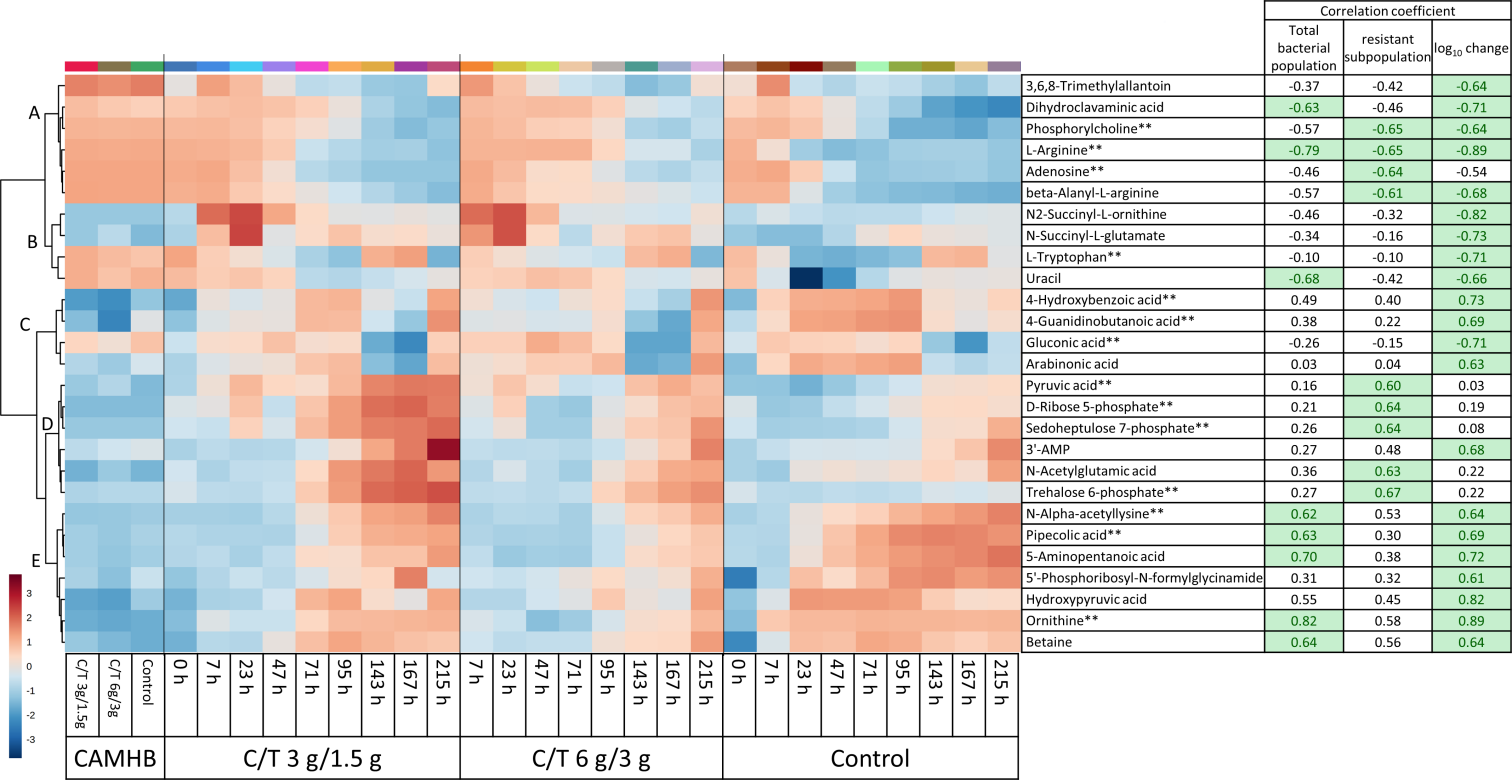
Heatmap with hierarchical clustering (**A–E**) of dynamic metabolite profiles, which correlated with the total bacterial population, resistant subpopulation, and/or the log_10_-change of the total population (<-0.6 or >0.6, shaded green) of *P. aeruginosa* CW41 challenged with ceftolozane-tazobactam (C/T) in a hollow-fiber infection model (*n* = 5). Metabolites notated with ** had MSI level 1 identification.

Of the 91 metabolites identified, 27 correlated (<−0.6 or >0.6) with one or more of the bacterial metrics (Fig. 2). The heatmap with HCA of these 27 metabolites revealed five clusters of interest. The first cluster of metabolomic footprints, denoted A in Fig. 2, decreased with increasing total bacterial density, and the next cluster (B, Fig. 2) of metabolites increased when the numerical value of the log_10_-change of the total population was the lowest (i.e., when there was the largest extent of bacterial killing between 7 and 47 h). The metabolites in these first two clusters were entirely negatively correlated with the bacterial metrics. In cluster B, uracil had an interesting profile, showing a vast uptake from the media in the control, but not in the drug-treated experimental arms. Conversely, N2-succinyl-ornithine and N-succinyl-glutamate peaked at 7–23 h, and correlated with the log_10_-change (Fig. 2), but were not as strongly correlated with the total bacterial population. Citrulline also showed this trend (Fig. S5), but the correlation coefficients fell below the threshold of the analyses.

In the cluster denoted C in Fig. 2, the metabolites only correlated with the log_10_-change of the total population. Interestingly, the next cluster (D), had five out of six clustered metabolites, which correlated exclusively with the resistant bacterial subpopulation. Of these five metabolites, three were in the pentose phosphate pathway (PPP; i.e., pyruvic acid, D-ribose-5-phosphate, and D-sedoheptulose-7-phosphate). The last cluster, denoted E, positively correlated with the total bacterial population and the numerical value of the log_10_-change. The pathways enriched with at least five correlating metabolomic footprints were arginine and proline metabolism, and central carbon metabolism (which includes the aforementioned PPP).

Based on the highest correlation coefficients per cluster, the enriched pathways, and manual filtering, the extracellular metabolites arginine, trehalose 6-phosphate, D-ribose 5-phosphate, D-sedoheptulose 7-phosphate, and ornithine were selected for mathematical modeling. The identity of each of these extracellular metabolites was confirmed by reference standards.

### Mathematical modeling

**Fig 3:**
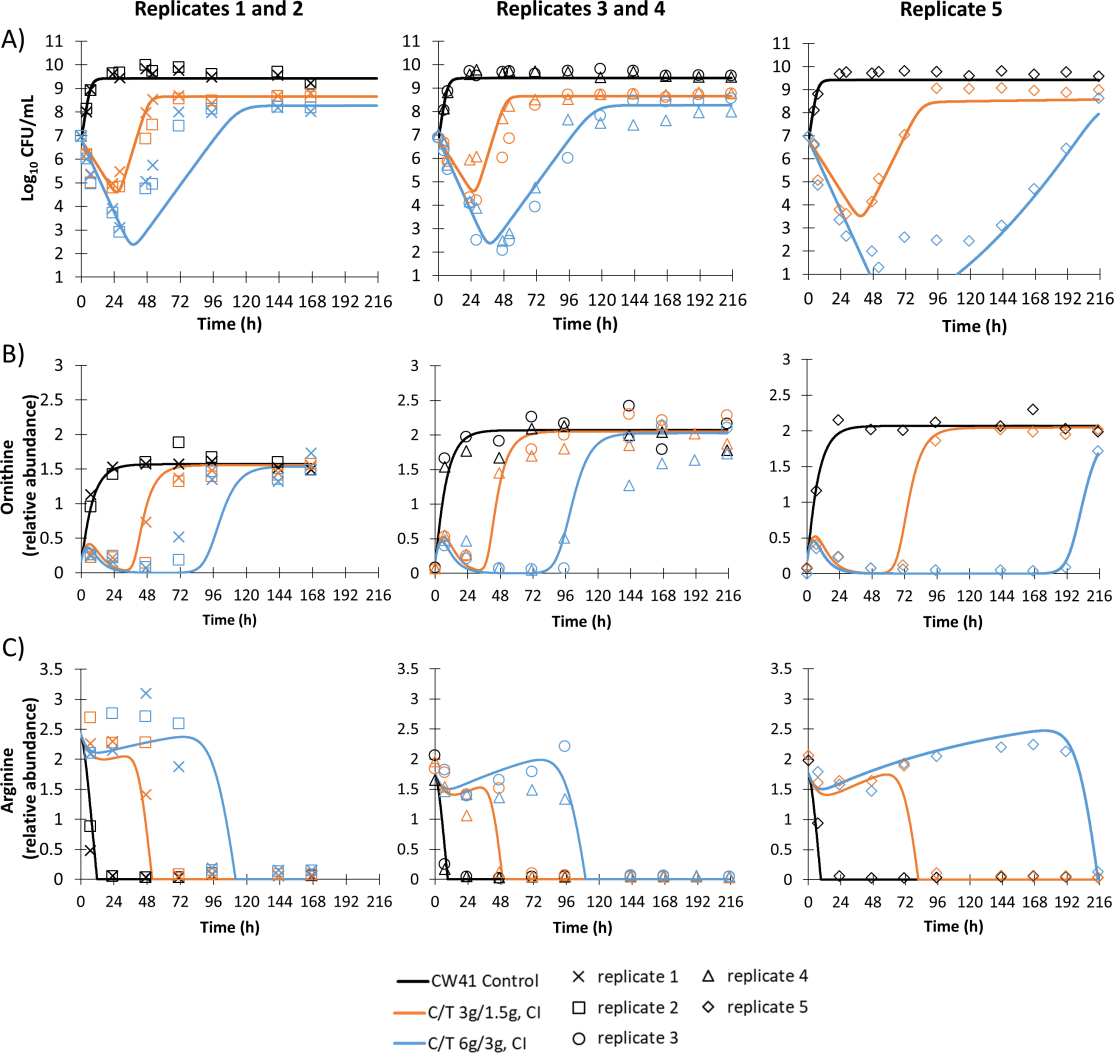
Population model fits from the mathematical modeling of ornithine (panel B) and arginine (panel C); extracellular metabolites related to the total bacterial population of *P. aeruginosa* CW41 (panel A) challenged with continuous infusion (CI) regimens of ceftolozane-tazobactam (C/T) in a hollow-fiber infection model (*n*=5).

**Fig 4:**
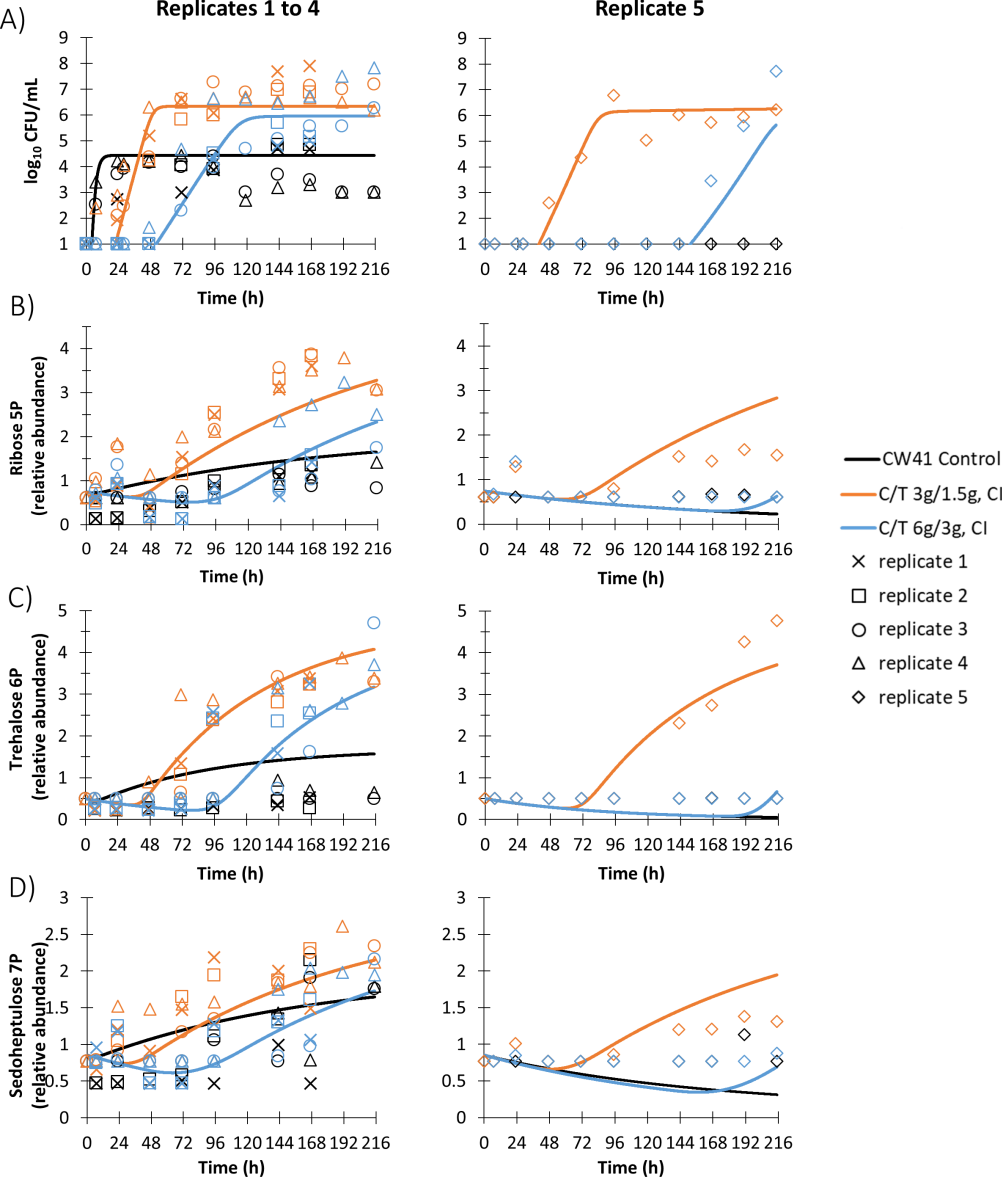
Population model fits from the mathematical modeling of D-ribose 5-phosphate (panel B), trehalose 6-phosphate (panel C), and D-sedoheptulose 7-phosphate (panel D); extracellular metabolites related to the resistant subpopulation of *P. aeruginosa* CW41 (panel A) challenged with continuous infusion (CI) regimens of ceftolozane-tazobactam (C/T) in a hollow-fiber infection model (*n* = 5).

Model-fitted extracellular ornithine and arginine in the HFIM are presented in Fig. 3B and C, and model-fitted extracellular D-ribose 5-phosphate, trehalose 6-phosphate, and D-sedoheptulose 7-phosphate in Fig. 4B through D. Diagnostic plots are presented in Fig. S6. Replicates 1–4 yielded reproducible profiles for the studied metabolites in the HFIM, in line with similar time-courses of the quantified bacterial populations reported previously [Fig. 3A and 4A; all of these replicates had observable pre-existing resistant subpopulations (8)]. By comparison, replicate 5 produced different metabolite profiles, which reflected the different time-courses of the bacterial populations that were observed in the HFIM due to a lack of observable pre-existing resistant subpopulations [(8), Fig. 3A and 4A]. The differences observed in the fifth replicate were described in the previously published bacterial model, and no extra model features were required to link the bacterial populations to any of the metabolites described here.

Early in the development of the mathematical models of the metabolites, alongside the final transduction model structure (Fig. 1), a biophase model structure was explored (Fig. S3), but this was discontinued as the runs in S-ADAPT did not reliably reach completion of the model run, and the AIC of the transduction model was more favorable (−89 versus −99, respectively).

The final transduction models and parameter estimates (Table 1) well described the selected extracellular metabolites and provided sufficiently precise fits for all five replicates, even without random variability between curves, as demonstrated in Fig. 3 and 4. These models had low relative bias within 20% (Fig. S6). Indeed, an interim model (estimated only based on study 1, replicates 1 and 2) was able to sufficiently predict the metabolite profiles of study 2 based on the respective bacterial profiles, even for the completely different bacterial and metabolite profiles of replicate 5, which were not used in the interim estimation (Fig. S7 to S11). For ornithine and arginine, the total bacterial population was used in the sigmoidal effect equation. The Hill coefficients for these extracellular metabolites were high (17.2 and 10.6, respectively). For the best fits, separate estimates for the maximal effect (*E*_MAX_) and initial metabolite level (*M*_INITIAL_) for studies 1 and 2 were required for ornithine and arginine, respectively (Table 1).

**TABLE 1:** Parameter estimates of the mathematical models describing the relationship between measured extracellular metabolites and the bacterial pharmacodynamic data of *P. aeruginosa* CW41 challenged with ceftolozane-tazobactam in the hollow-fiber infection model^*a*^

Parameter	Symbol (unit)	Parameter estimates (SE%)				
		Ornithine	Arginine	D-ribose 5-phosphate	D-sedo-heptulose 7-phosphate	Trehalose 6-phosphate
Bacterial input	(log_10_ CFU/mL)	CFU_ALL_	CFU_ALL_	CFUonDP_20_	CFUonDP_20_	CFUonDP_20_
Baseline, for inhibitory effect	*E*_0_	−	2.96 (12.4%)	−	−	−
Maximum effect	*E*_MAX_ (relative abundance)	1.57 (6.9%)^*b*^, 2.07 (6%)^	41.1 (13%)	9.62 (21.6%)	6.13 (28.9%)	5.66 (15.7%)
Bacterial density causing 50% of maximum effect	EC_50_ (log_10_ CFU/mL)	6.67 (4.6%)	7.51 (4.9%)	6.27 (14.8%)	6.76 (31.9%)	5.01 (13.1%)
Hill coefficient	Hill	17.2 (15.1%)	10.6 (21%)	3.72 (23.2%)	1.76 (36%)	6.92 (7.4%)
Transition time to and from the metabolite compartment	*τ* (h)	7.82 (21.3%)	141 (8.1%)	179 (11.1%)	165 (16%)	90.5 (21.1%)
Initial conditions of differential equation for metabolite	*M*_INITIAL_ (relative abundance)	0.17 (34.4%)	2.42 (7.7%)^*b*^, 1.77 (5.7%)^	0.744 (29%)	0.853 (6.4%)	0.497 (26.5%)
SD of residual error	SD_MLITE_	0.145 (8.3%)	0.128 (13%)	0.342 (9.1%)	0.289 (7.2%)	0.383 (8.3%)

^*a*^ CFU_ALL_, total bacterial population quantified on antibiotic-free CAMHA; CFUonDP_20_, resistant bacterial subpopulation quantified on CAMHA containing ceftolozane-tazobactam (20 mg/L).

^*b*^ Estimates for study 1 (replicates 1 and 2), ^estimates for study 2 (replicates 3, 4, and 5).

For D-ribose 5-phosphate, D-sedoheptulose 7-phosphate, and trehalose 6-phosphate, the transduction models with the resistant bacterial subpopulation well described the measured extracellular metabolites, particularly replicates 1–4 (Fig. 4). In the fifth replicate, D-ribose 5-phosphate and D-sedoheptulose 7-phosphate were slightly overpredicted from 143 h onward (Fig. 4). Trehalose 6-phosphate was slightly underpredicted at 191 and 215 h (Fig. 4).

## DISCUSSION

The future of optimizing antibiotic treatment requires moving away from time-collapsed and point-based measures and toward time-course-based analysis of patient–drug–pathogen interactions (2, 39). TDM is a well-established approach to account for variability in the PK component of the patient–drug interactions (4). Markers, such as procalcitonin and other inflammatory markers, provide information on patient–pathogen interactions over time (40). However, a marker to measure drug–pathogen interactions (such as bacterial killing and emergence of resistance) over time is not yet available. Here, we explored whether extracellular metabolites could be used as markers to monitor bacterial density and resistant regrowth in the *in vitro* HFIM, with a hypermutable MDR *P. aeruginosa* clinical isolate challenged with ceftolozane-tazobactam via continuous infusion.

The uptake of arginine and secretion of ornithine were highly correlated with the total bacterial density in the HFIM. In aerobic conditions, such as those in the HFIM, arginine, and ornithine, uptake is facilitated by transporters coded on the *aot* operon that are involved with the arginine succinyltransferase pathway, which utilizes both these metabolites as carbon sources (41, 42). Anaerobic uptake of arginine (a nitrogen source in this condition) occurs via the ArcD antiporter with simultaneous secretion of ornithine and is associated with the arginine deiminase pathway (43). This pathway aids anaerobic cellular respiration, which is an essential process for *P. aeruginosa* to thrive in the anoxic conditions of respiratory infections of patients with CF (44). The arginine deiminase pathway regulator, ANR, has been found in aerobic *in vitro* culture previously; thus, it is possible that ANR and thus ArcD were active in the aerobic HFIM (45–47). Otherwise, readily secreted ornithine could indicate auxotrophic growth with regard to arginine, as fresh nutrient-rich media is constantly added in the HFIM (48, 49). Intracellular metabolomics or proteomics would be required to elucidate the underlying mechanisms and ultimately fall outside the scope of these studies. Nevertheless, the uptake of arginine and secretion of ornithine in aerobic *in vitro* conditions has been reported previously (50–52), albeit in studies performed over a much shorter timeframe (10–24 h) in static batch culture and were not the main focus of that research. In the current studies, the mathematical modeling of ornithine and arginine was completed with the total population as the bacterial input. Hill coefficients were high, likely arising from differences between seemingly small changes in bacterial populations on log-scale and the large changes of metabolites on normal scale. Importantly, the differences between replicates 1–4 and the fifth replicate in the profiles of these metabolites were well described using the bacterial mechanism-based mathematical model we presented previously (8), and no adjustments were required in the metabolomic mathematical model for the fifth replicate.

Interestingly, D-ribose 5-phosphate and D-sedoheptulose 7-phosphate were elevated in extracellular samples of the drug treatment arms in the second half of each HFIM run and correlated with the resistant bacterial subpopulation. If these PPP metabolites were in the extracellular media as a result of bacterial killing by ceftolozane-tazobactam, it would be expected that they would peak when there was a large decrease in bacterial density (53). Peak concentrations of citrulline, N2-succinyl-ornithine, and N-succinyl-glutamate occurred at 7–23 h when log_10_-change was the absolute greatest, but this did not occur for D-ribose 5-phosphate and D-sedoheptulose 7-phosphate. Alternatively, the PPP may have been upregulated in the resistant regrowth due to oxidative stress, since the mechanism of bacterial killing by β-lactams involves elevated levels of reactive oxygen species (ROS) prior to cell death (54, 55). To produce reducing agents to neutralize ROS, the enzyme glucose-6-phosphate dehydrogenase (G6PD) in the entryway to the PPP can be upregulated (56, 57). Upregulation of G6PD and subsequent flux into the PPP would not be limited by depletion of carbon sources, such as glucose or glycerol, considering the constant inflow of media in the HFIM. D-Ribose 5-phosphate and D-sedoheptulose 7-phosphate may have been secreted as metabolic overflow if the amount produced from the PPP was in excess of that required for purine and lipopolysaccharide biosynthesis, respectively (58). Additionally, purine synthesis may have been suppressed in the resistant regrowth to lower the lethality of ceftolozane-tazobactam (59).

Similar to D-ribose 5-phosphate and D-sedoheptulose 7-phosphate, extracellular trehalose 6-phosphate was elevated after exposure to ceftolozane-tazobactam and correlated with resistant growth. In the literature, intracellular trehalose 6-phosphate in *P. aeruginosa* was also elevated after exposure to polymyxin B (60). The de-phosphorylated form, trehalose, can serve as a carbon source but also has an important role in bacteria during osmotic stress (61, 62). The presence of extracellular trehalose has been found to be significantly associated with MDR *P. aeruginosa* isolates, compared to susceptible isolates (63). Ultimately, the signaling pathways that regulate trehalose (and thus trehalose 6-phosphate) in osmotic and other stress-response pathways are currently undefined (62). The detected extracellular trehalose 6-phosphate was likely a result of a combination of metabolic stress under antibiotic pressure and subsequent metabolism overflow from the resistant bacteria in the HFIM, as with the metabolites in the PPP.

The mathematical models based on resistance slightly over- or underpredicted D-ribose 5-phosphate, D-sedoheptulose 7-phosphate, and trehalose 6-phosphate in the fifth replicate. This replicate was more challenging for the models as a resistant bacterial subpopulation was not detected in the control, and correspondingly, metabolite concentrations (and thus calculated relative abundance) were below and therefore fixed to the minimum detected peak intensity (100,000) prior to relative abundance calculations. However, the observed general trend of elevated metabolite concentrations by the low-dose ceftolozane-tazobactam treatment in the fifth replicate was clearly apparent in all three models of the metabolites, which correlated with resistance (Fig. 4). Long estimated transition times in these three metabolites (Table 1) helped to achieve good population fits of the shape of the curves, while shorter transition times would have resulted in fitted curves that were too steep (and thus overpredicted the observed metabolite levels earlier in the time-course).

The classic PK/PD transduction model was able to describe the metabolomic data for all five metabolites in relation to the total bacterial population and resistant subpopulations in the HFIM. While some transition times were long (but within the duration of the HFIM runs), adding more theoretical compartments between the effect and the selected metabolite might predict shorter transition times, but would be no more descriptive of the presented data set. In addition, the estimated time required for transition out of the metabolite compartment in the model encompassed the re-uptake of secreted metabolites by the bacteria, as well as metabolite washout from the circulating media in the cartridge and central reservoir to the waste.

These studies being the first to dynamically profile extracellular metabolites of *P. aeruginosa* in the HFIM is a major strength of this research, particularly considering the robust data produced across five biological replicates. We used a hypermutable MDR strain of *P. aeruginosa*, which is a near-worst case scenario for a patient, and a new β-lactam/β-lactamase inhibitor combination. Untargeted metabolomic footprint analysis revealed five metabolites that related to total bacterial density and, importantly, resistant regrowth for this clinically important combination of pathogen and antibiotic. Monitoring resistance over time is pertinent to *P. aeruginosa*, which has an exceptional ability for resistance emergence during antibiotic exposure, even to novel antibiotics (64). Most metabolomic studies to date employed static culture conditions (13). A relatively recent study used a one-compartment *in vitro* infection model (65), the setup of which includes a continuous loss of bacteria with diluting media. In contrast, the HFIM is an infection model with contained bacteria and diluting media. The HFIM was suitable for the dynamic metabolite footprint analyses reported here since these analyses do not rely on a constantly high bacterial density, unlike intracellular (i.e., fingerprint) metabolomics that require high inocula with large sample volumes (e.g., 15 mL of 8 log_10_CFU/mL). A limitation of these studies is the generalizability of the noted relationships between the time-courses of the concentrations of metabolites and the bacterial density of the *P. aeruginosa* isolate treated with ceftolozane-tazobactam in the HFIM. Considering the high time and cost requirements of conducting HFIM and subsequent metabolomic analyses, a single isolate treated with one antibiotic in five biological replicates was deemed suitable for these proof-of-concept studies. The sequence of preferred nutrient uptake may vary between *P. aeruginosa* isolates (50). Thus, the metabolomic findings presented here warrant further investigation with other *P. aeruginosa* isolates, including reference strains, e.g., PAO1, non-hypermutable strains, and eventually with other β-lactam antibiotics (53). The five metabolites that were described with mathematical modeling in these studies are not exclusive to bacterial metabolism. Indeed, they are ubiquitous in mammalian metabolism as well. Therefore, these modeled metabolites are not likely to be suitable biomarkers for monitoring antibiotic treatment response *in vivo*. However, the approach demonstrated in this paper could be utilized to identify bacteria-specific metabolites that potentially may be more specific for *in vivo* monitoring. In an ideal scenario with a robust and bacteria-specific metabolite*,* a monitoring marker that correlated with resistance emergence could potentially be used to facilitate early detection of treatment failure in patients.

## Data Availability

The figures and tables include the data from the reported studies. A datafile of the relative abundances of 91 identified extracellular metabolites in the hollow-fiber infection model is available for download. The studies are available at the NIH Common Fund’s National Metabolomics Data Repository (NMDR) website, the Metabolomics Workbench, https://www.metabolomicsworkbench.org. Study 1 (with replicates 1 and 2) has been assigned Study ID ST003036, and study 2 (replicates 3, 4 and 5), ST003024.

## SUPPLEMENTAL MATERIAL

Supplemental figures and tables aac.01081-23-s0001.docx Additional model qualification and results of metabolomic data analysis.

Metabolomics dataset aac.01081-23-s0002.xlsx Relative abundances of extracellular metabolites identified in hollow-fiber infection model of *P. aeruginosa* CW41 versus ceftolozane/tazobactam.

ASM does not own the copyrights to Supplemental Material that may be linked to, or accessed through, an article. The authors have granted ASM a non-exclusive, world-wide license to publish the Supplemental Material files. Please contact the corresponding author directly for reuse.
