# Supplementary material for: Identifying and mathematically modeling the time-course of extracellular metabolic markers associated with resistance to ceftolozane/tazobactam in Pseudomonas aeruginosa
Source: Antimicrob Agents Chemother. 2024 Feb 20;68(4):e01081-23. doi: 10.1128/aac.01081-23 (PMC10989016; doi:10.1128/aac.01081-23)
Supplement: Supplemental figures and tables — Additional model qualification and results of metabolomic data analysis. [file aac.01081-23-s0001.docx]

## **Supplementary material**

# Identifying and mathematically modeling the time-course of extracellular metabolic markers associated with resistance to ceftolozane/tazobactam in *Pseudomonas aeruginosa*

Jessica R. Tait, Dovile Anderson, Roger L. Nation, Darren J. Creek, Cornelia B. Landersdorfer^#^

## ^#^Address correspondence to: cornelia.landersdorfer@monash.edu

**Table S1** Data precision represented as the median relative standard deviation (RSD) for 91 extracellular metabolites of *P. aeruginosa* CW41 measured in the hollow-fiber infection model.

| Time (h) | Median RSD (%)^ | | |
| --- | --- | --- | --- |
|  | Control | C/T  3 g/1.5 g | C/T  6 g/3 g |
| 0 | 9 | 9 |  |
| 7 | 11 | 16 | 13 |
| 23 | 15 | 18 | 19 |
| 47 | 15 | 21 | 18 |
| 71 | 14 | 24 | 20 |
| 95 | 15 | 24 | 23 |
| 143 | 17 | 18 | 33 |
| 167 | 20 | 17 | 29 |
| 215 | 16 | 19 | 14 |
| bacteria-free CAMHB | 26 |  |  |
| PQC | 16 |  |  |
| ^n=3 for 0 and 215 h. n =5 from 7 h to 167 h. n=33 for CAMHB. n=18 for PQC. C/T ceftolozane-tazobactam | | | |


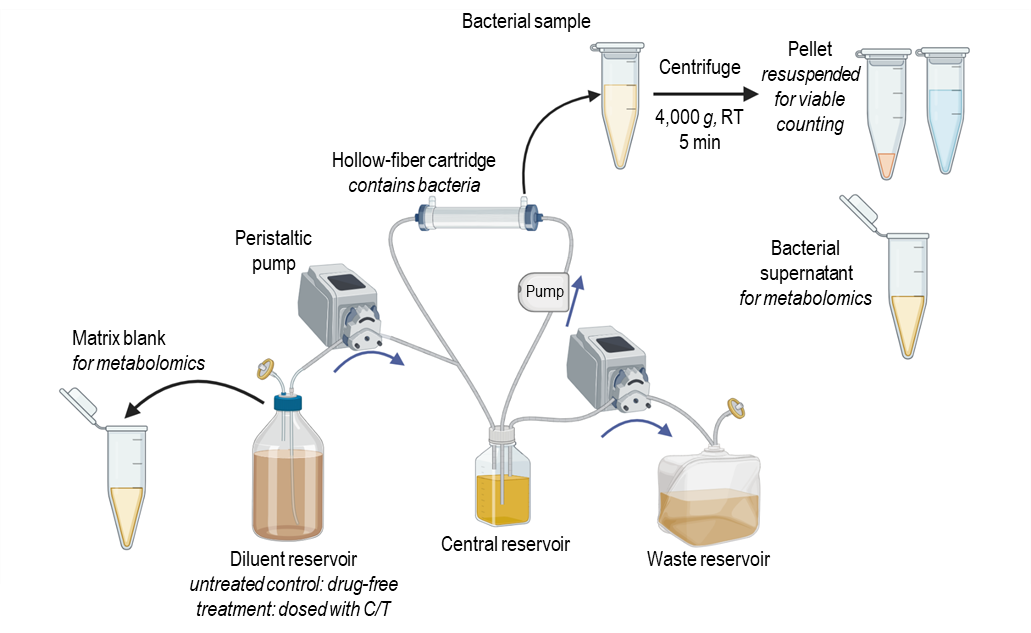


**Figure S1** Schematic of the hollow-fiber infection model and origin of the bacterial samples for viability counting, and the bacterial supernatant and matrix blank samples for metabolomics


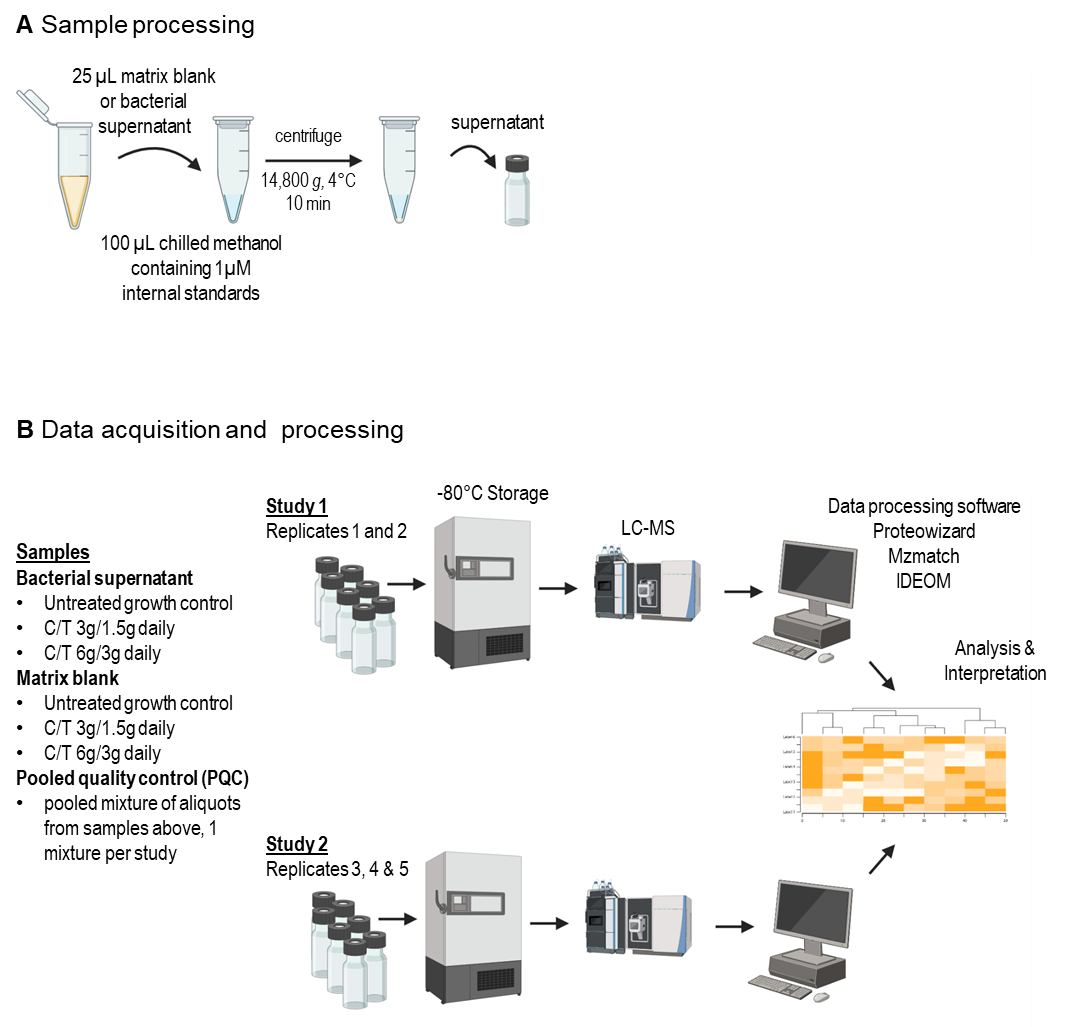


**Figure S2** Diagram of (A) sample processing for metabolomic analysis and (B) overview of data acquisition and processing workflow including list of samples


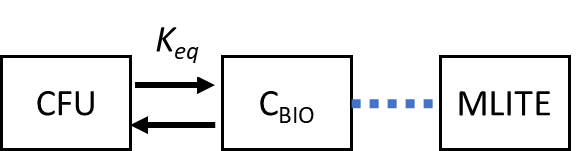


**Figure S3** Schematic of the biophase model investigated during model development to translate between bacterial data (CFU) and metabolite data (MLITE), *via* estimation of a theoretical biological process or compound (C_BIO_), which determined the metabolite level *via* an E_max_ effect equation.


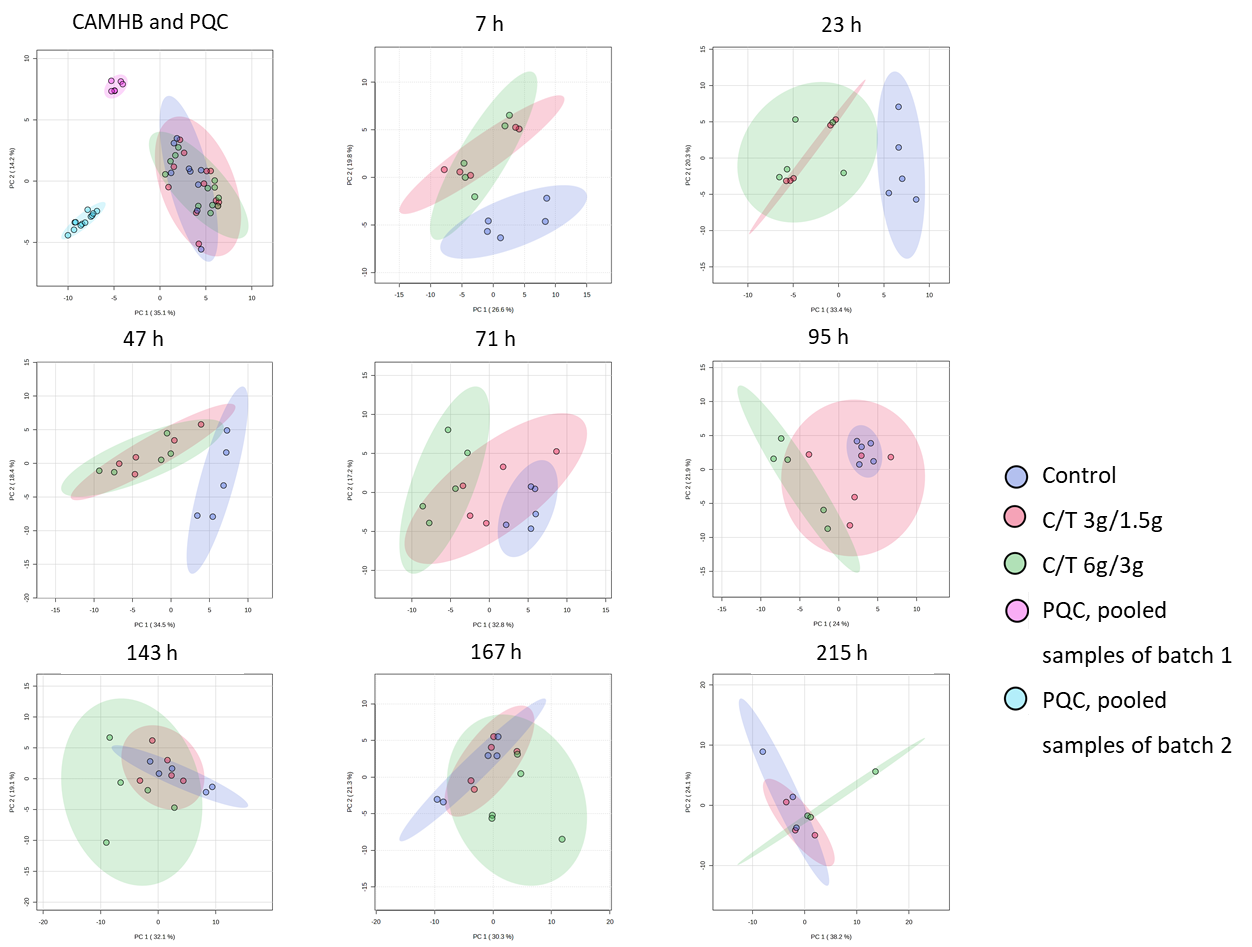


**Figure S4** Principal component analysis (PCA) plot of dynamic extracellular metabolomic profiles of *P. aeruginosa* CW41 challenged with ceftolozane-tazobactam (C/T) in the hollow-fiber infection model (n=5), plotted per timepoint. Pooled quality control (PQC) samples were plotted with the bacteria-free cation adjusted Mueller Hinton broth (CAMHB) samples.


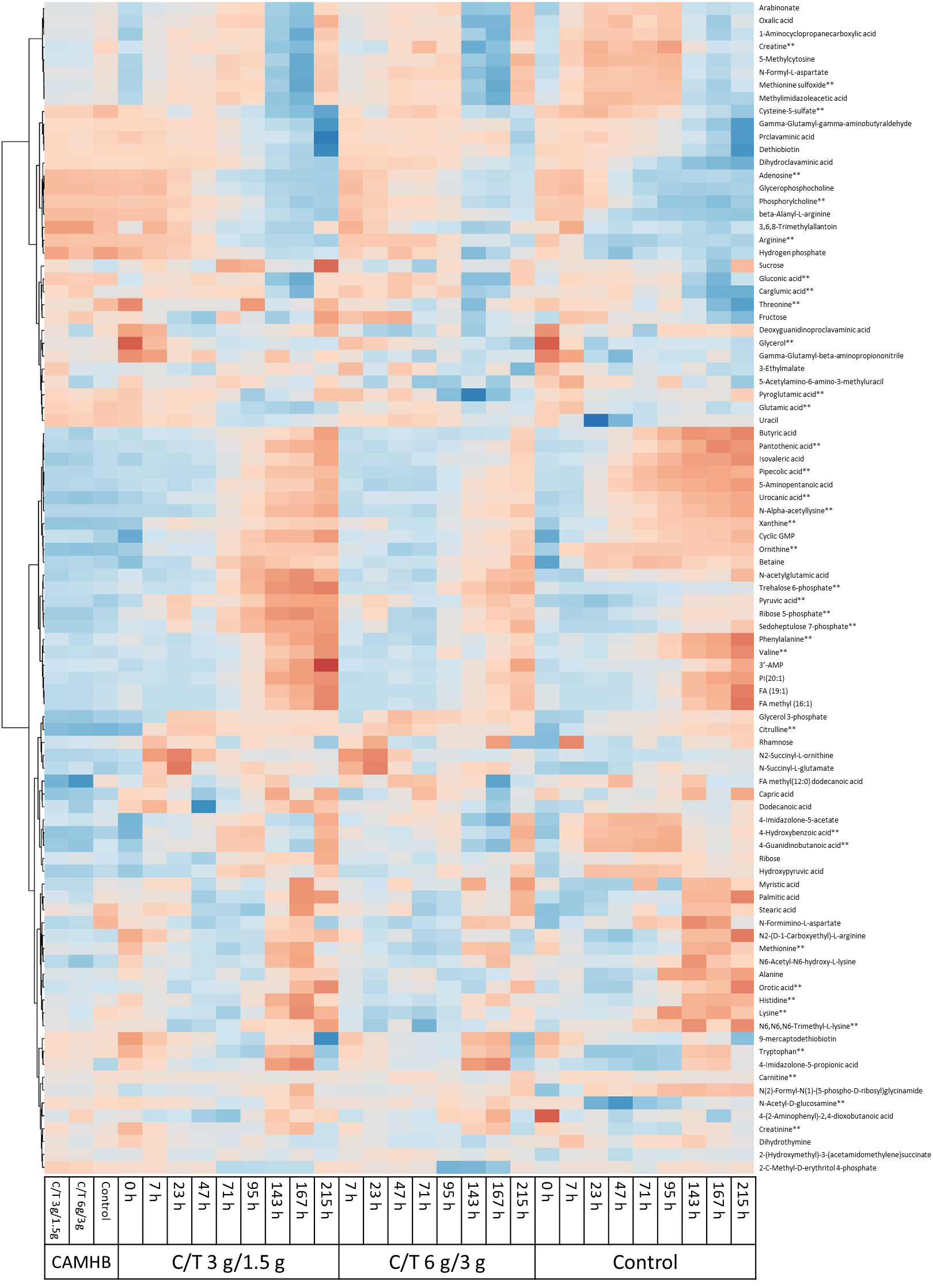
**Figure S5** Hierarchical clustering and heatmap of 91 extracellular metabolites of
*P. aeruginosa* clinical isolate CW41 challenged with ceftolozane-tazobactam (C/T) in the hollow-fiber infection model. Metabolites notated with ** had MSI level 1 identification. See the uploaded dataset for a detailed presentation

**Figure S6** Diagnostic plots (including observed vs individual (DV vs iPRED), observed vs population (DV vs PRED), and the expected weighted residual (EWRES vs TIME) of the 5 modeled metabolites in the HFIM. The relative bias (rBias) and relative root mean squared error (rRMSE) are displayed.

|  | DV vs iPRED | DV vs PRED | EWRES vs TIME |
| --- | --- | --- | --- |
| Ornithine  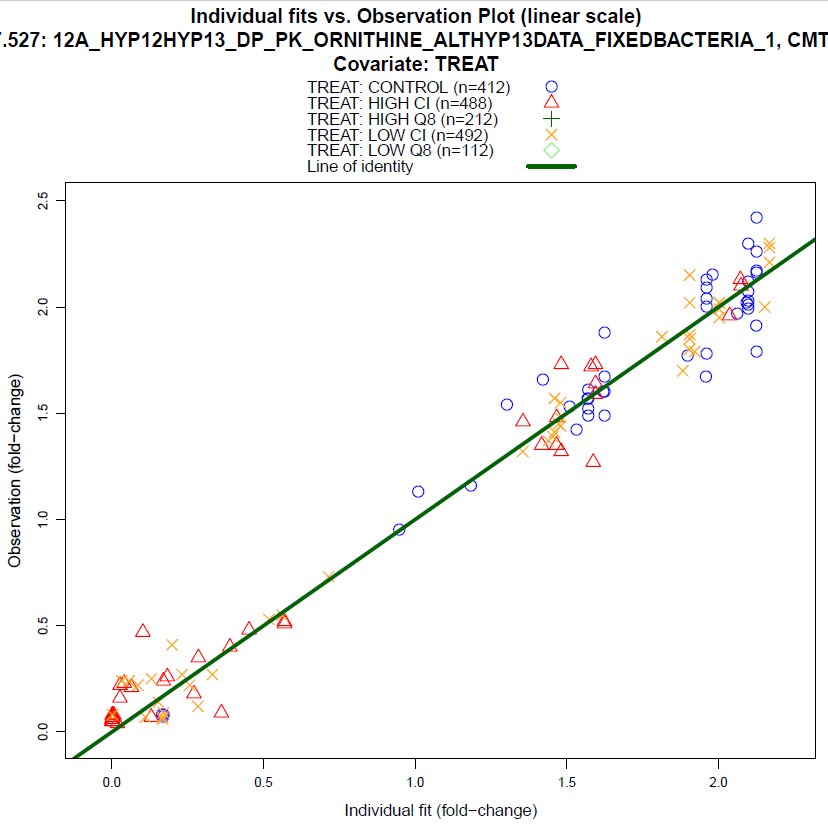Control  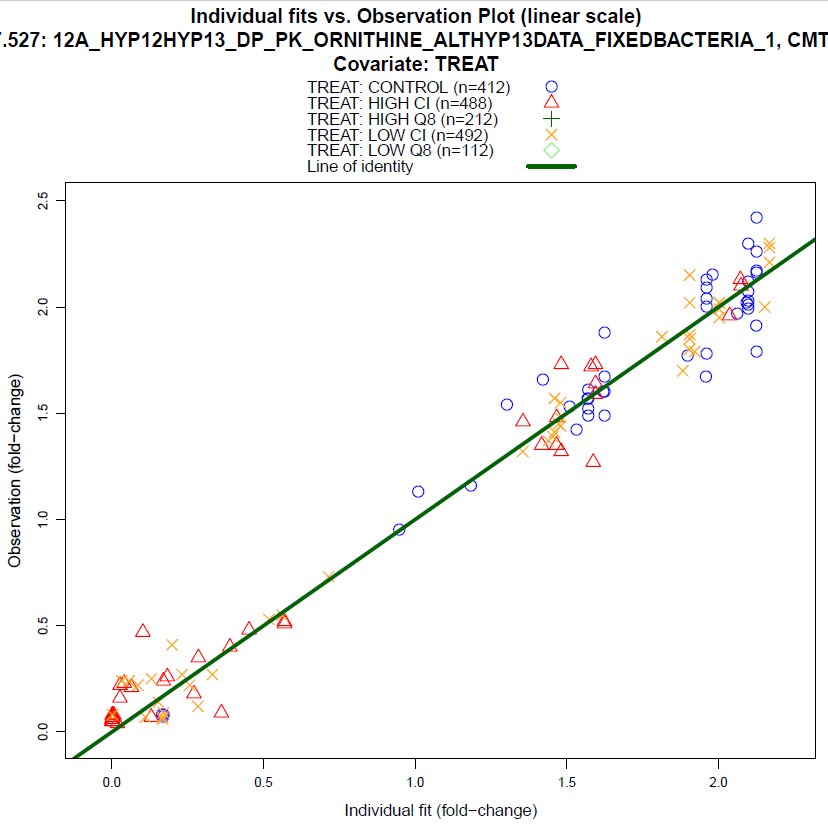3g/1.5g C/T  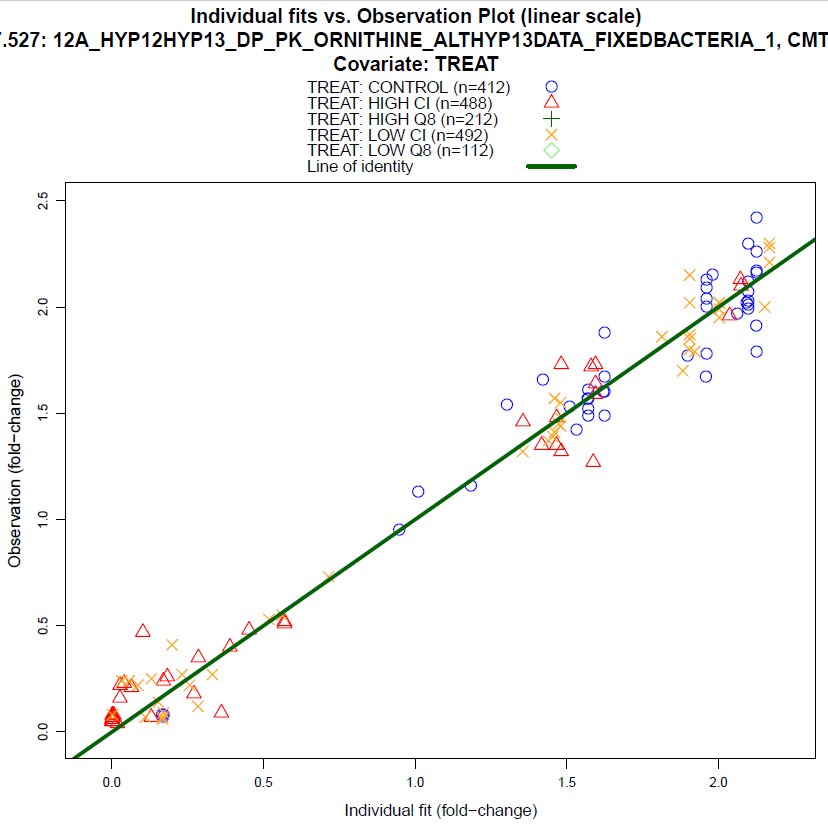6g/3g C/T | 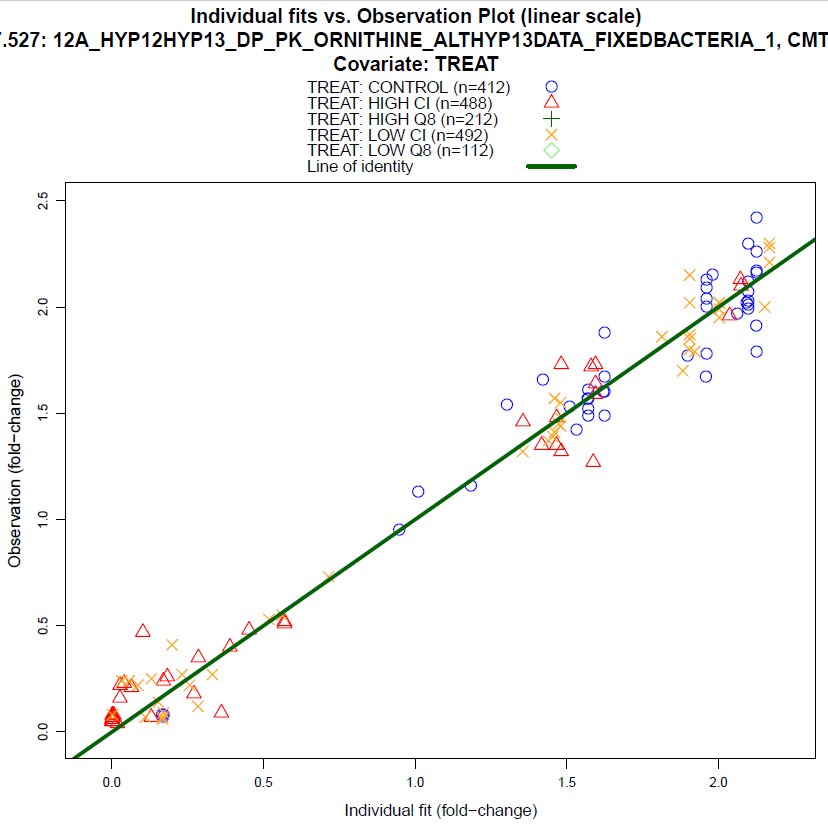  Observed data (relative abundance)  Individual fit (relative abundance)  r^2^ = 0.98  rBias = 7%  rRMSE= 70% | 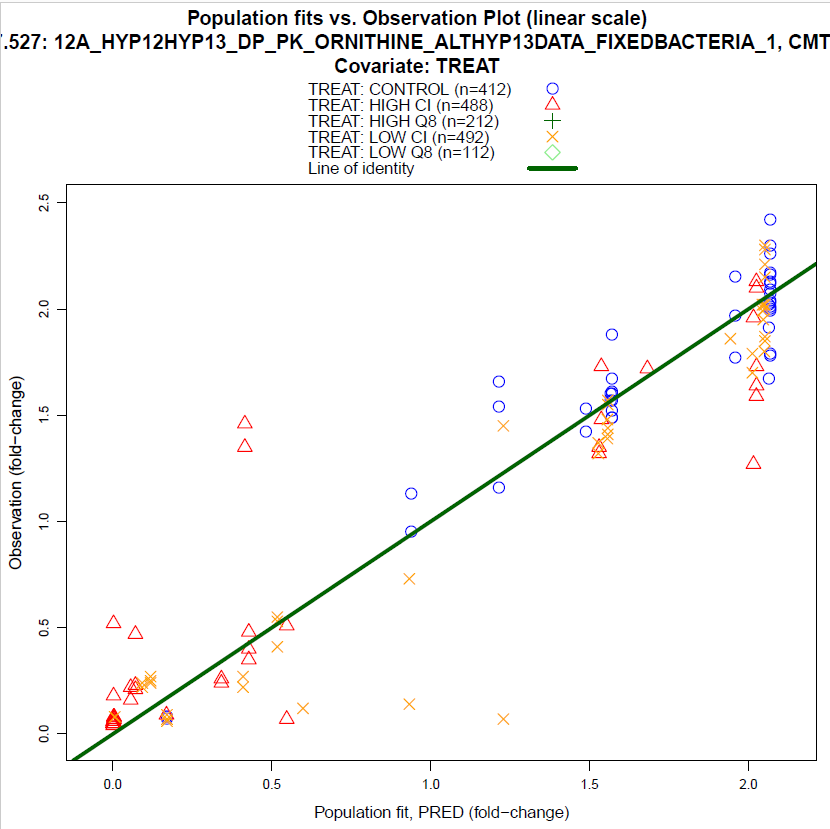  population fit (relative abundance)  Observed data (relative abundance)  r^2^ = 0.90  rBias = 10%  rRMSE= 79% | 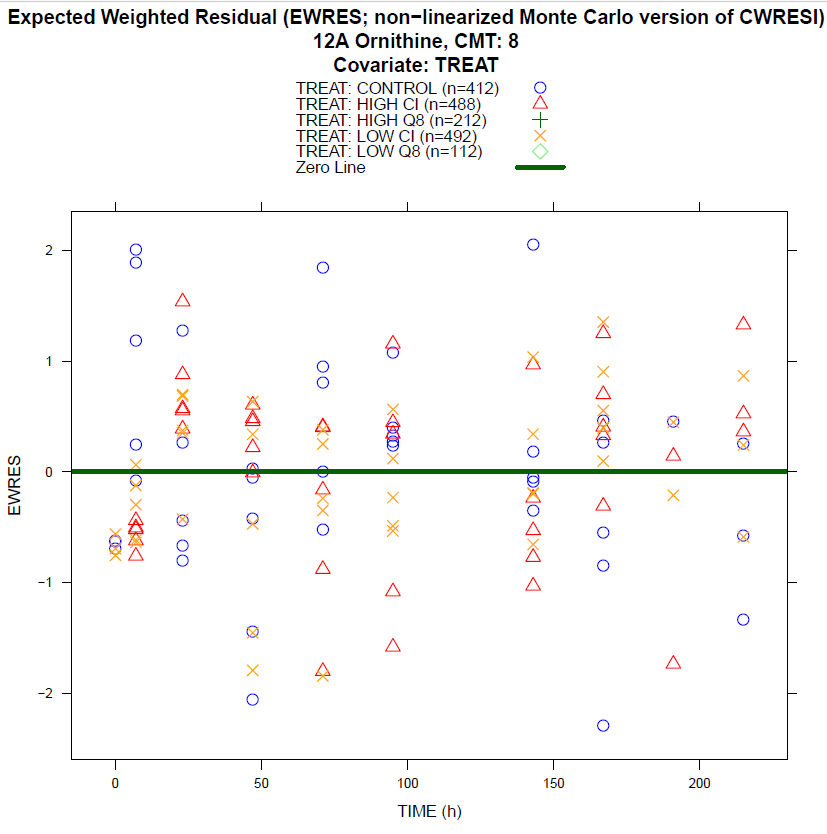 |
| Arginine  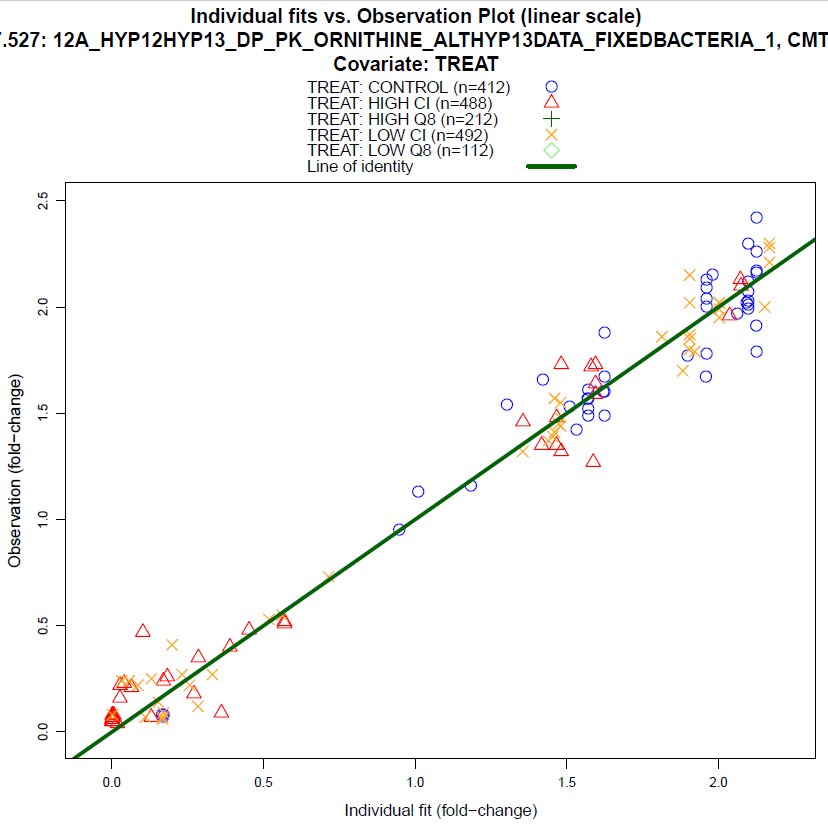Control  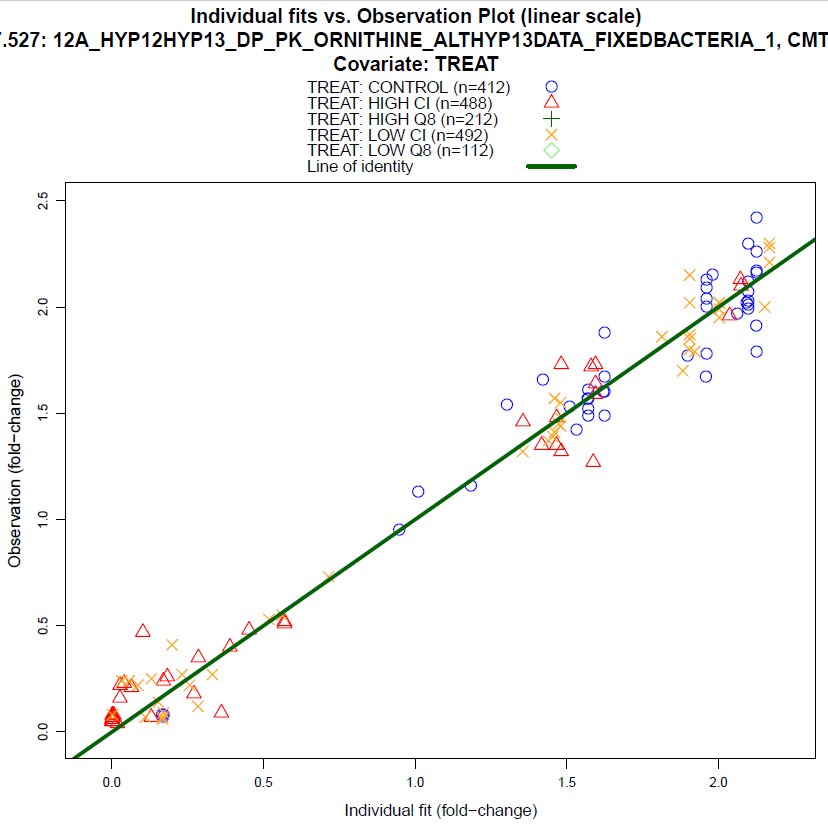3g/1.5g C/T  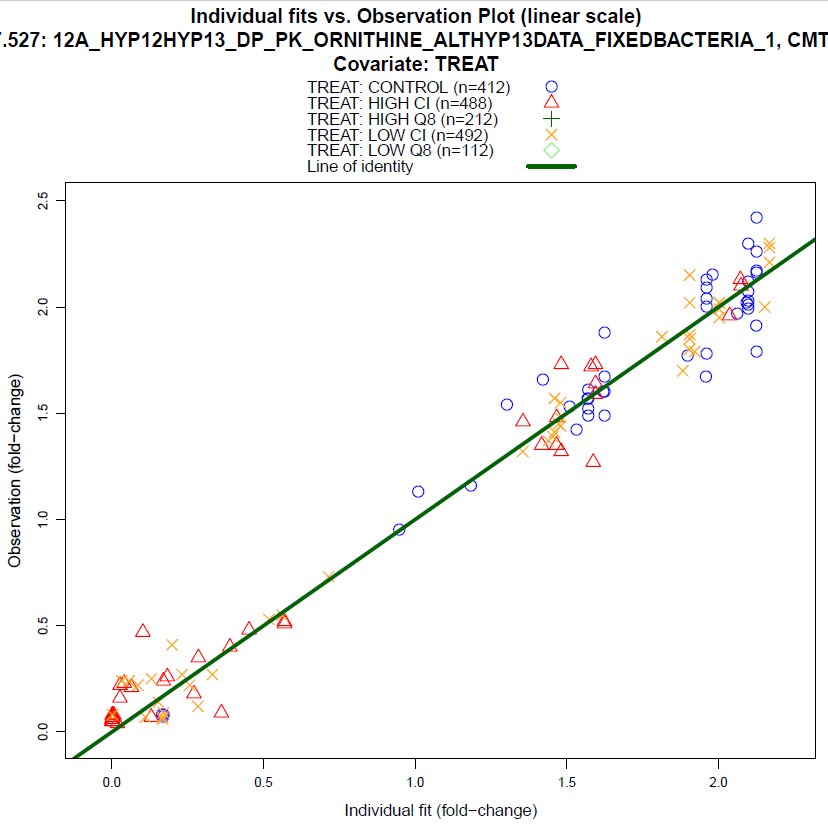6g/3g C/T | 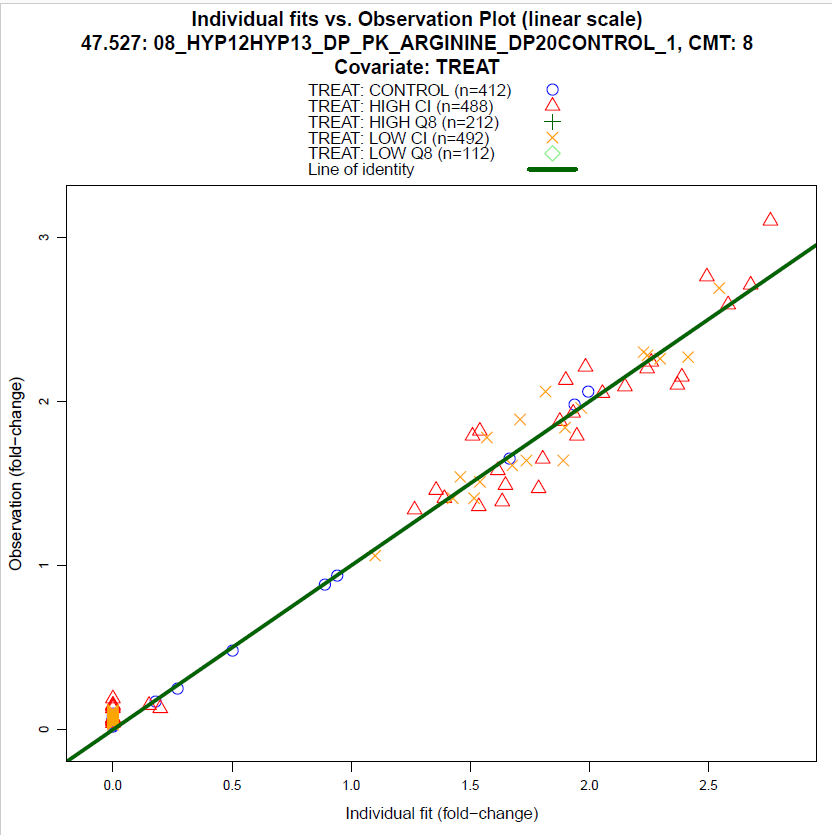  Individual fit (relative abundance)  Observed data (relative abundance)  r^2^ = 0.99  rBias = -83%  rRMSE= 151% | 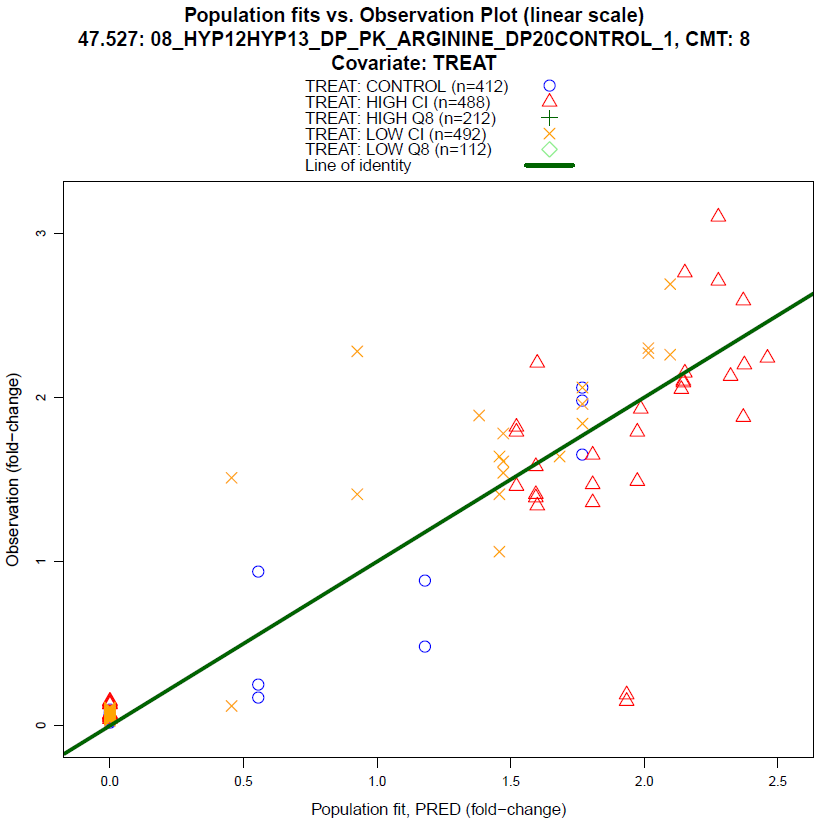  population fit (relative abundance)  Observed data (relative abundance)  r^2^ = 0.86  rBias = -81%  rRMSE= 151% | 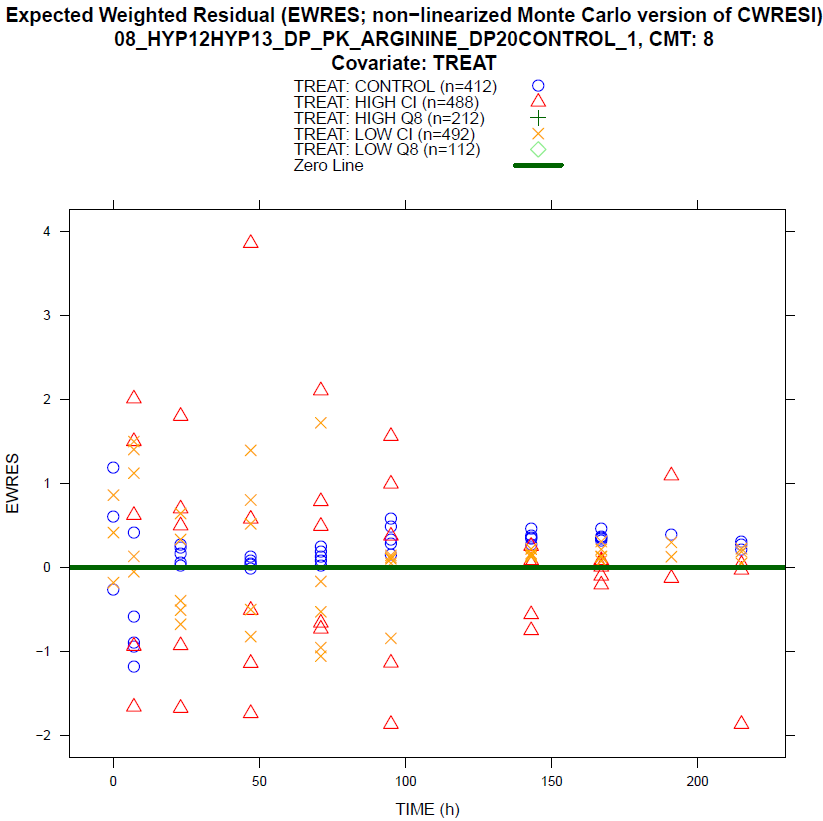 |
| D-Ribose 5P  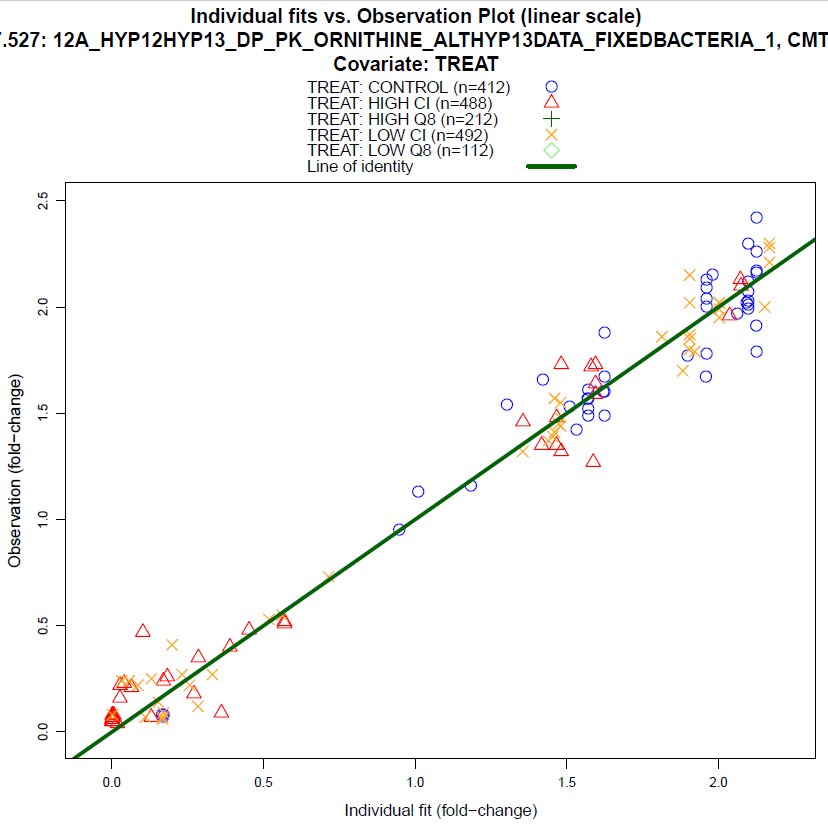Control  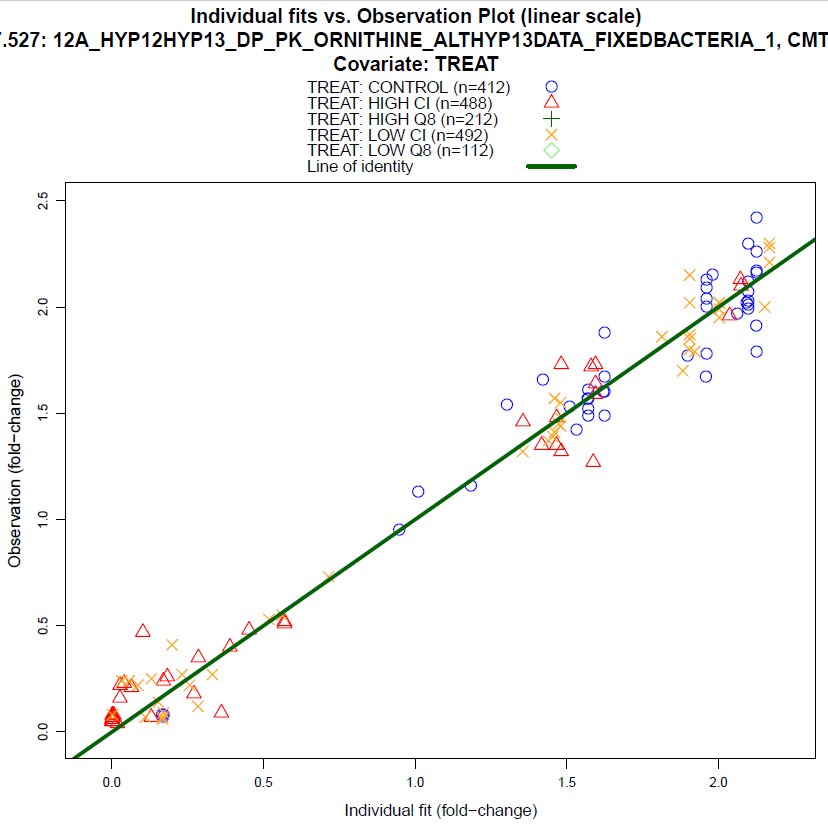3g/1.5g C/T  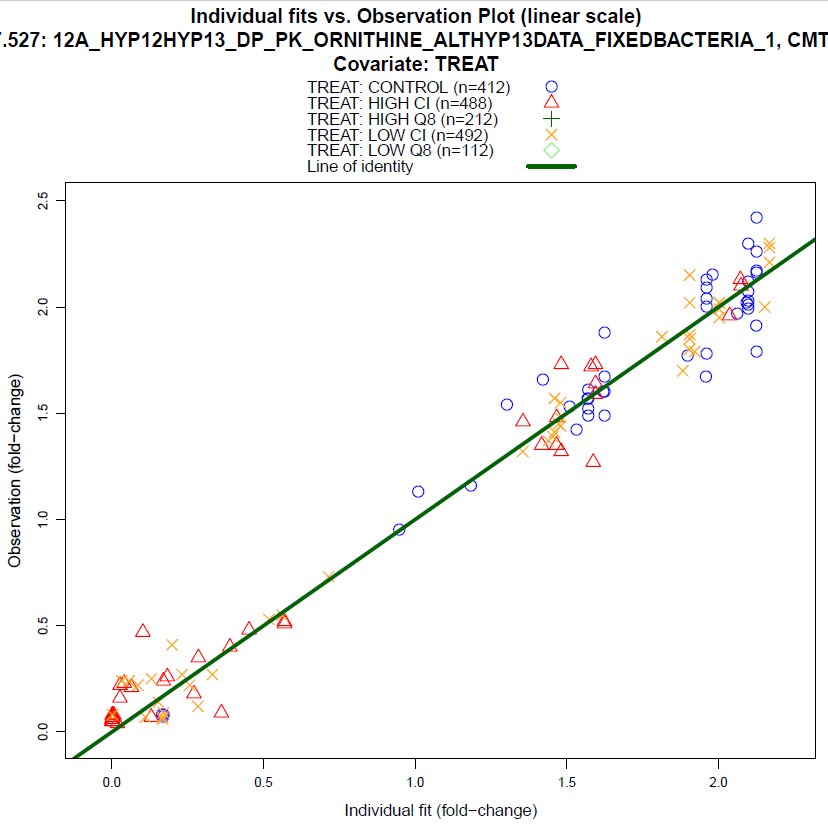6g/3g C/T  NB: the clusters at 0.13 and 0.61 relative abundance are the lower limits of detection for study 1 and 2, respectively. | 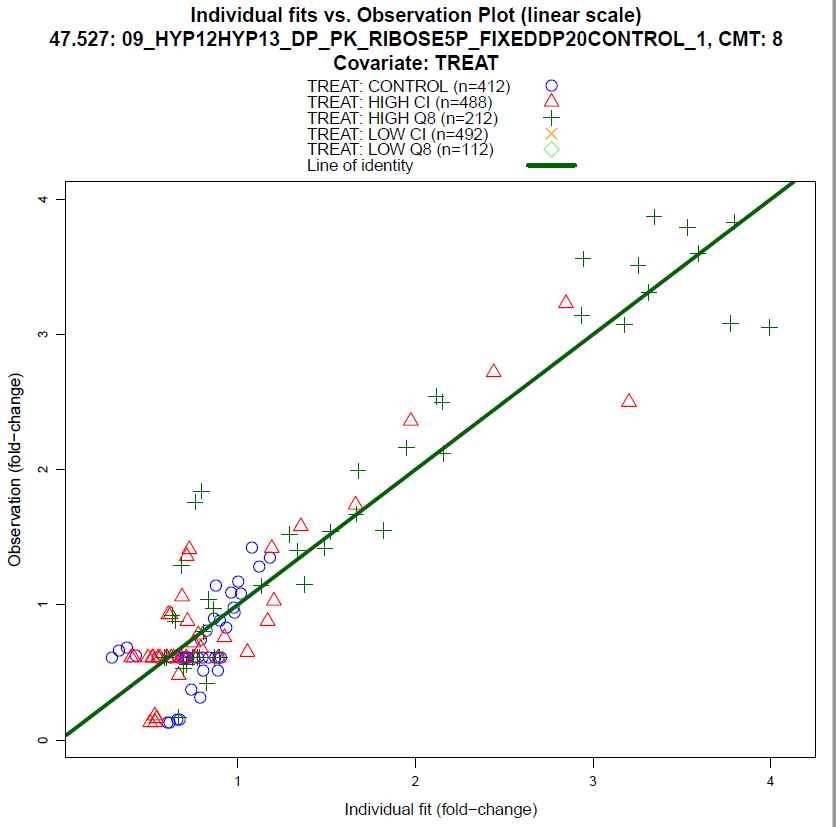  Individual fit (relative abundance)  Observed data (relative abundance)  r^2^ = 0.88  rBias = 19%  rRMSE= 43% | 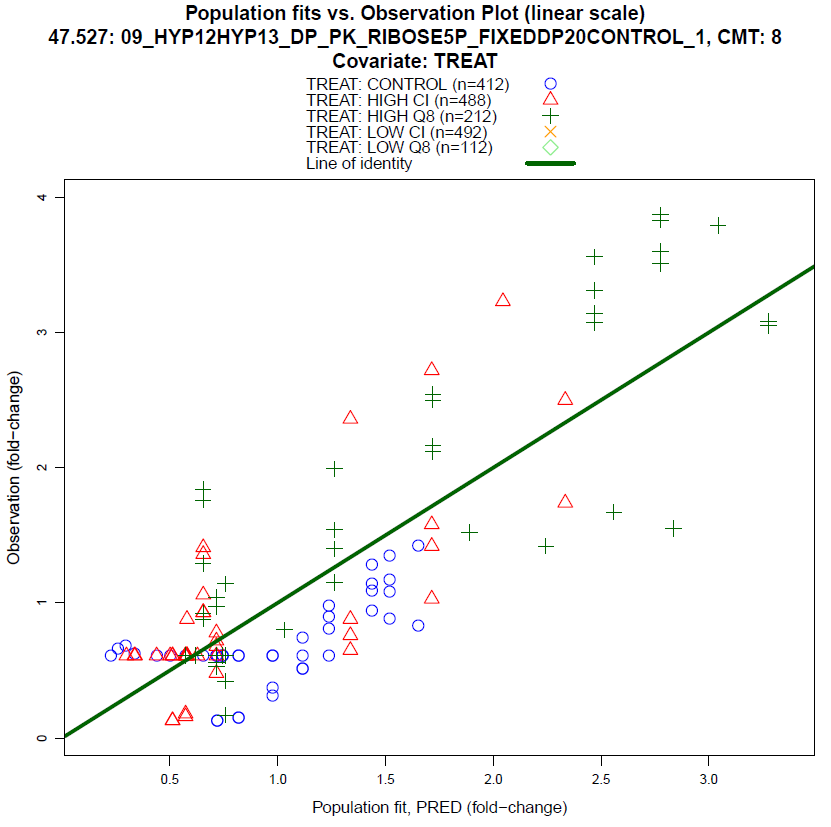  population fit (relative abundance)  r^2^ = 0.70  rBias = 18%  rRMSE= 52%  Observed data (relative abundance) | 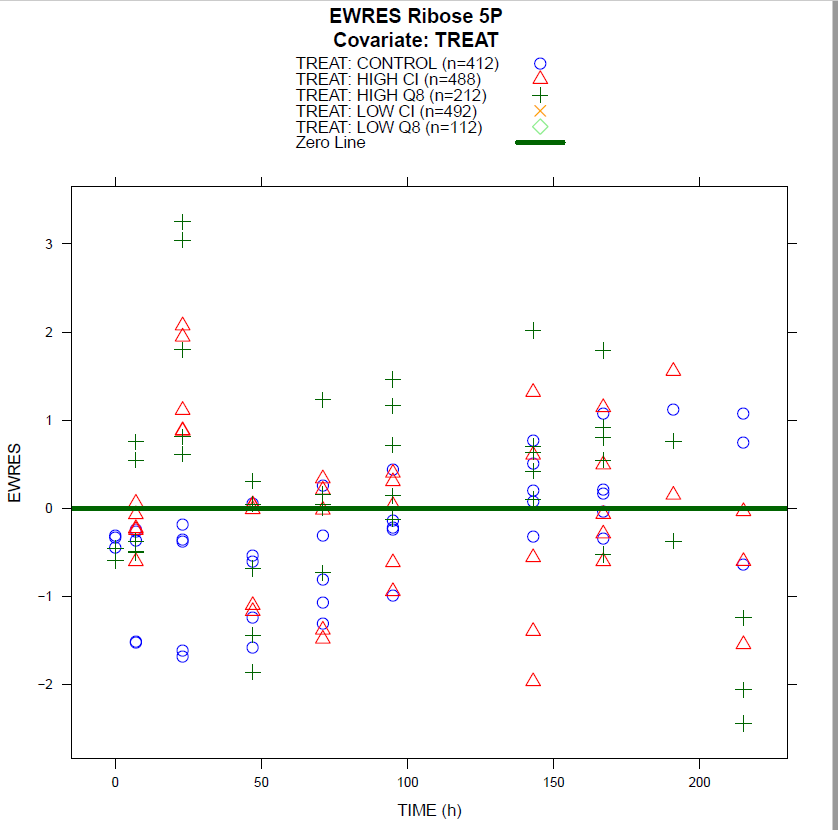 |
| Sedoheptulose 7P  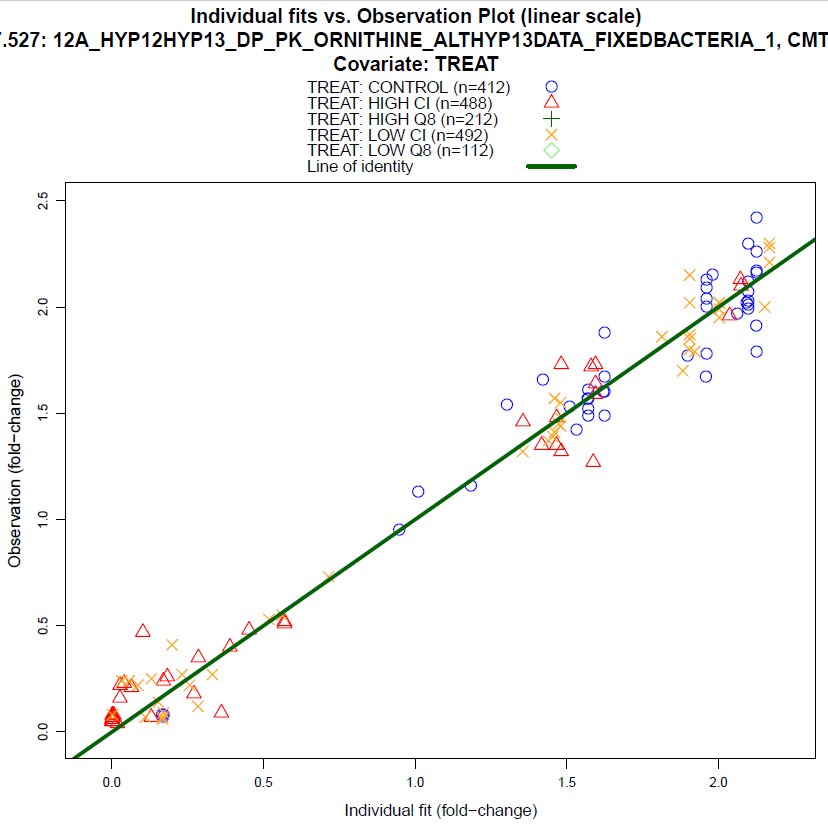Control  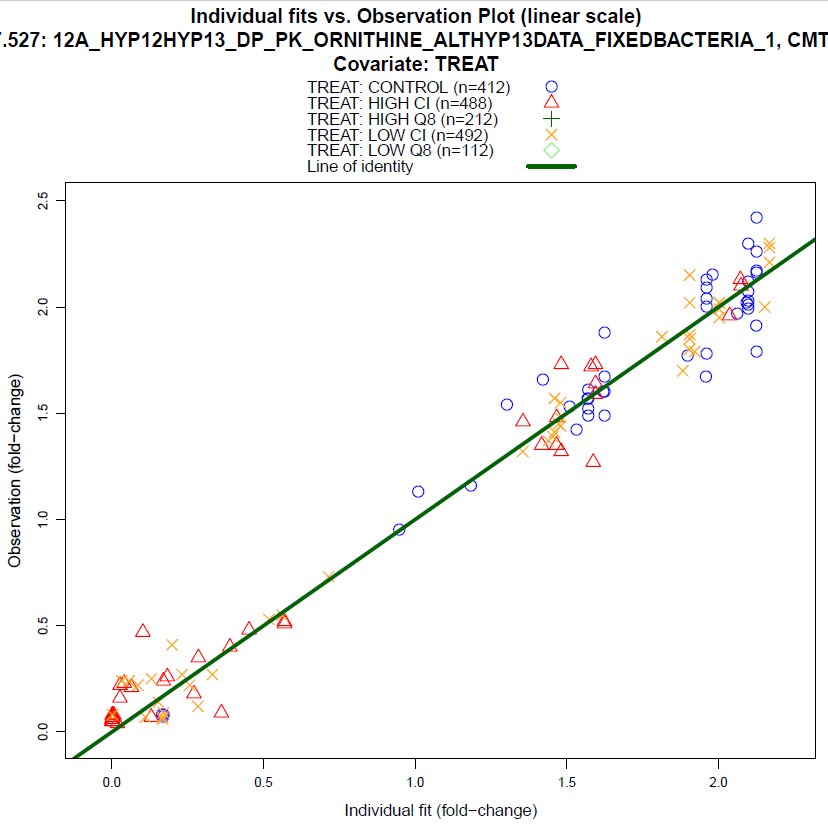3g/1.5g C/T  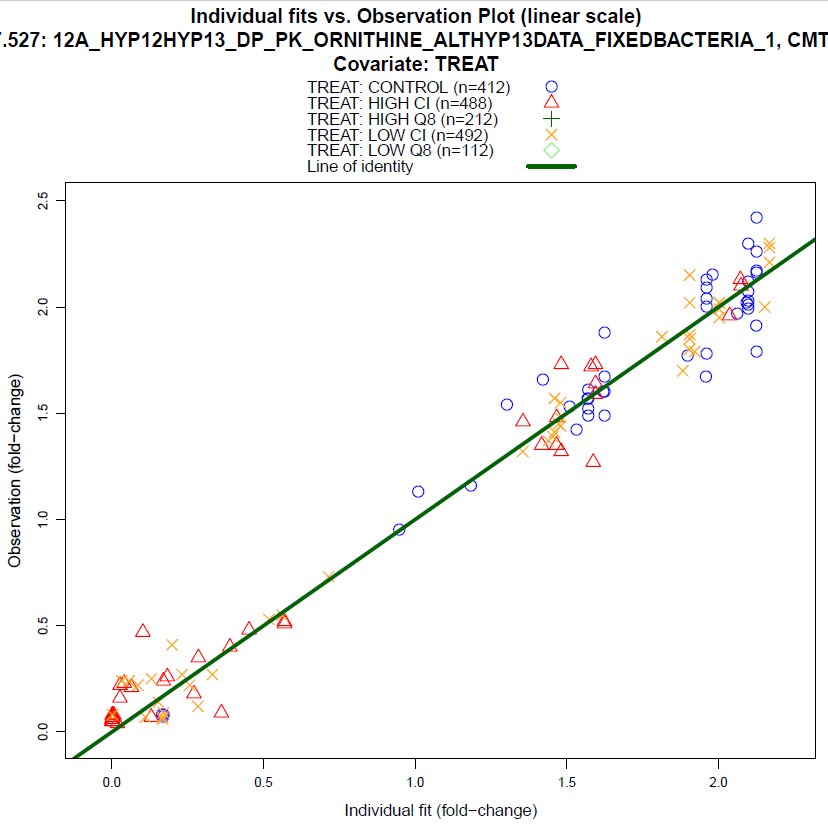6g/3g C/T  NB: the clusters at 0.47 and 0.77 relative abundance are the lower limits of detection for study 1 and 2, respectively. | 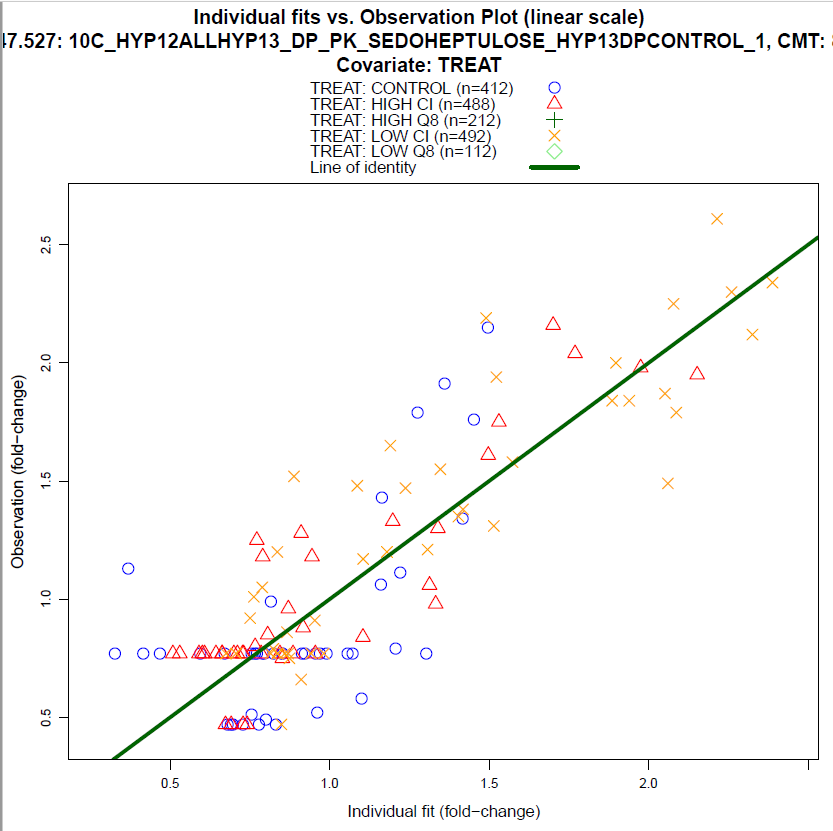  Individual fit (relative abundance)  Observed data (relative abundance)  r^2^ = 0.73  rBias = 7%  rRMSE= 29% | 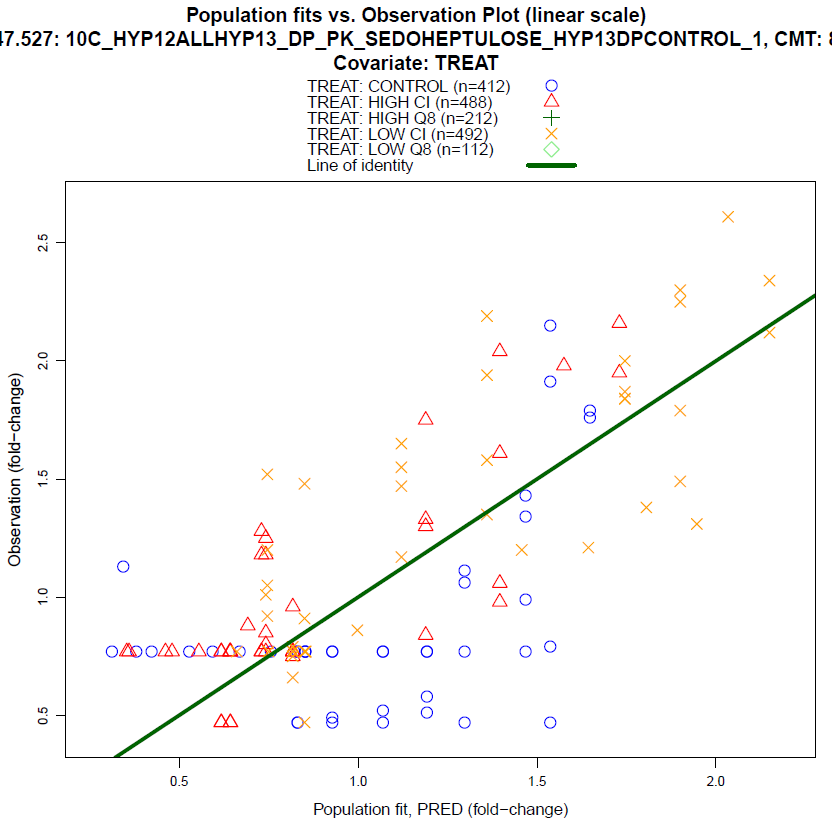  population fit (relative abundance)  Observed data (relative abundance)  r^2^ = 0.52  rBias = 6%  rRMSE= 38% | 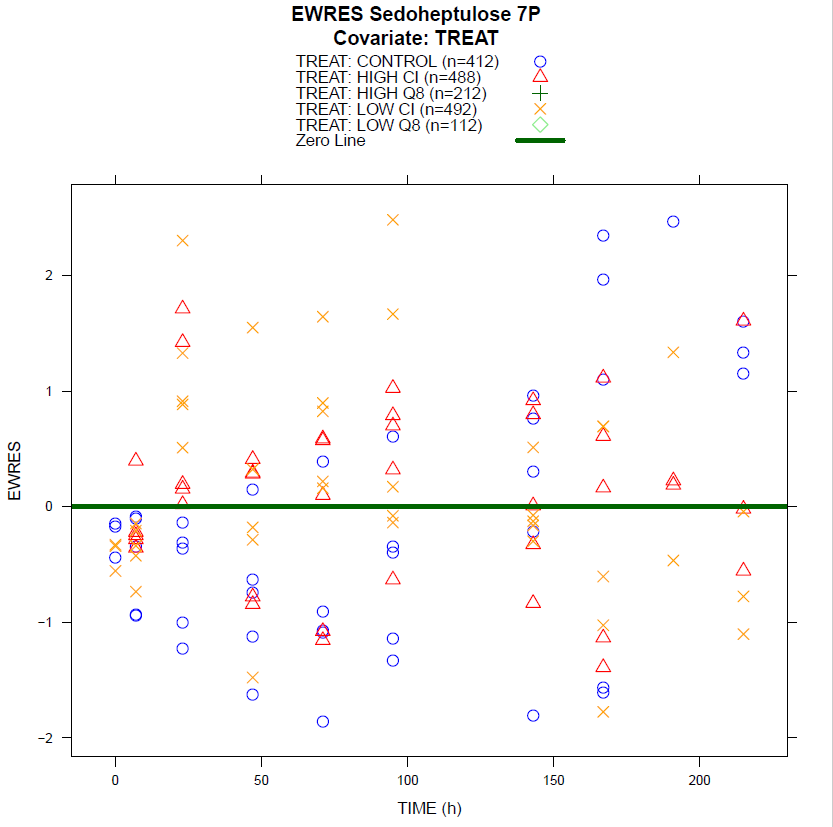 |
| Trehalose 6P  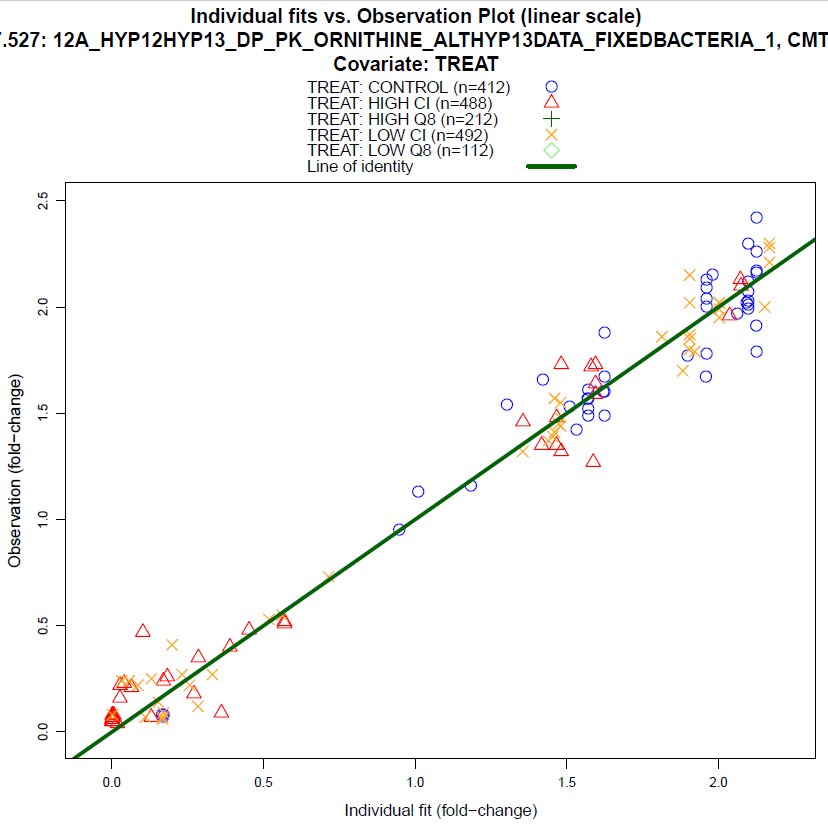Control  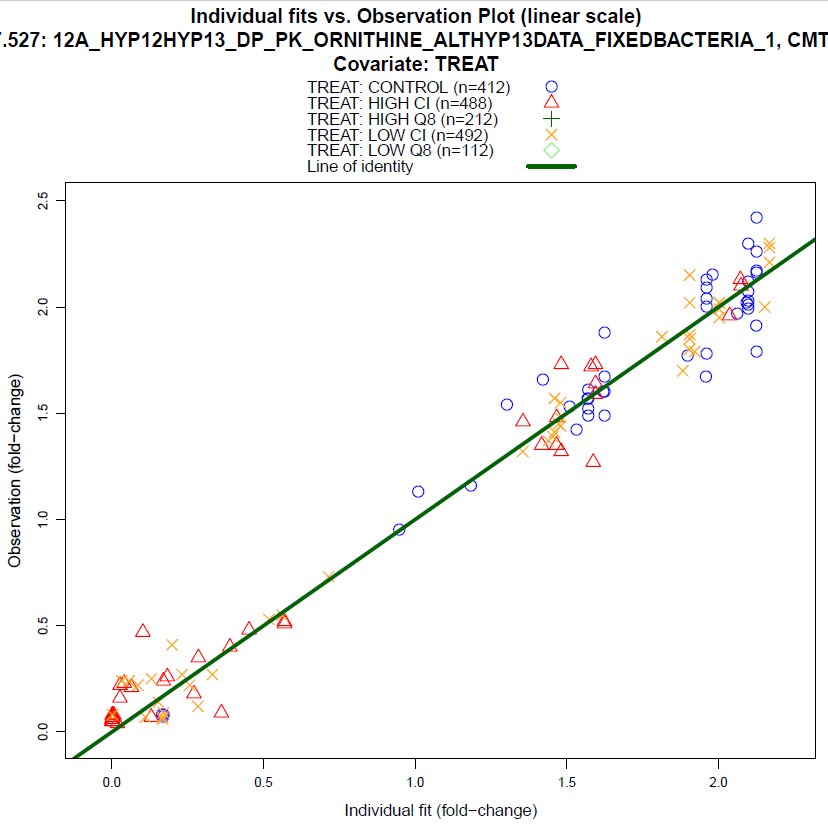3g/1.5g C/T  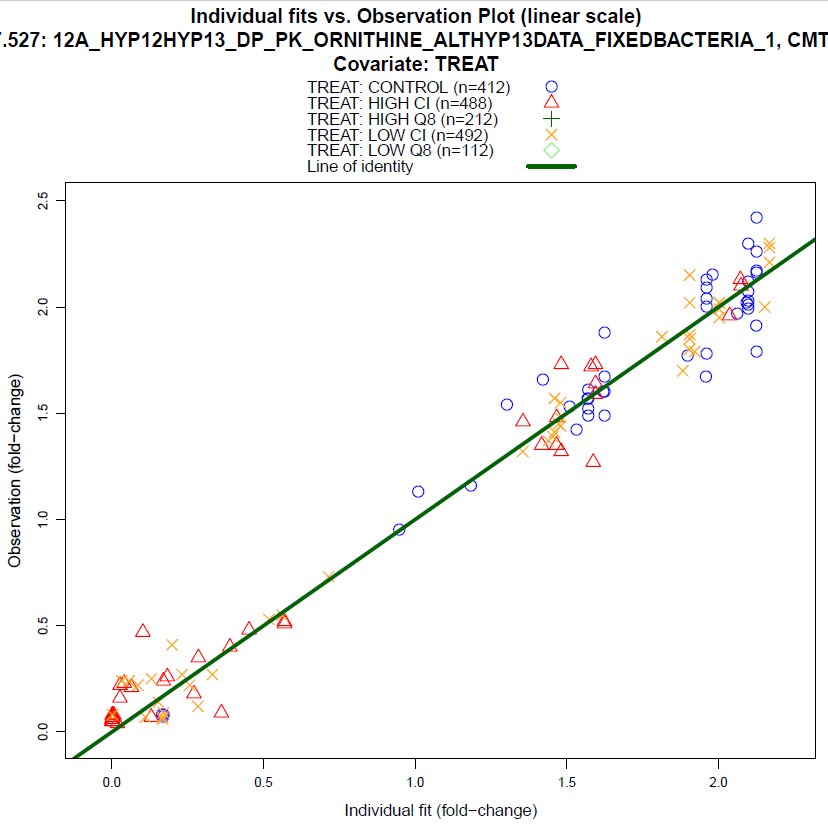6g/3g C/T  NB: the clusters at 0.22 and 0.50 relative abundance are the lower limits of detection for study 1 and 2, respectively. | 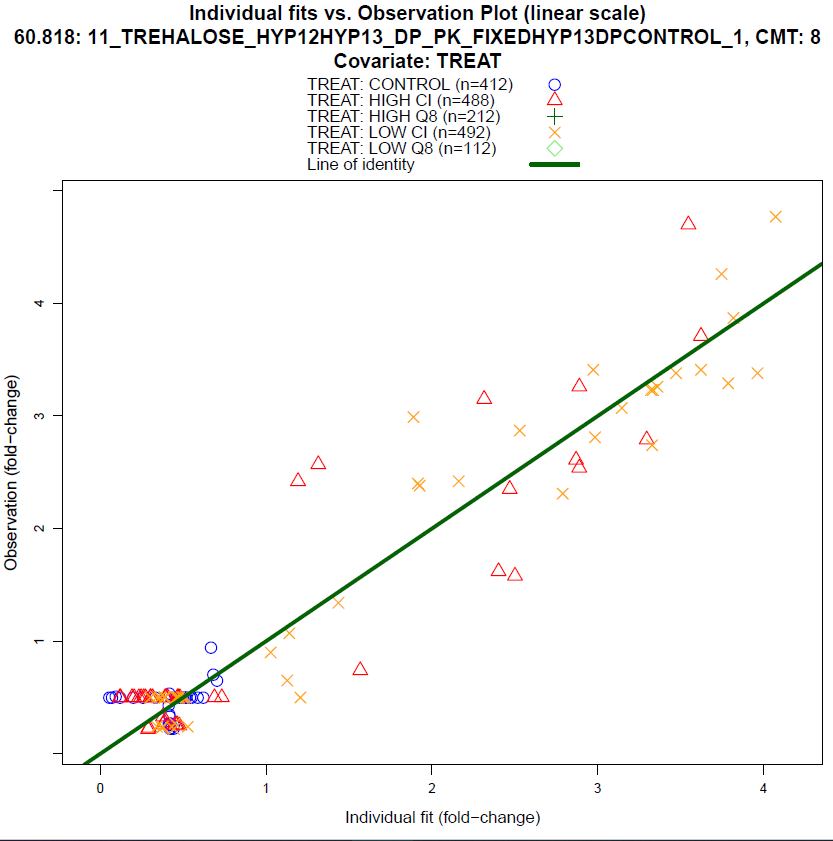  Individual fit (relative abundance)  Observed data (relative abundance)  r^2^ = 0.91  rBias = 7%  rRMSE= 47% | 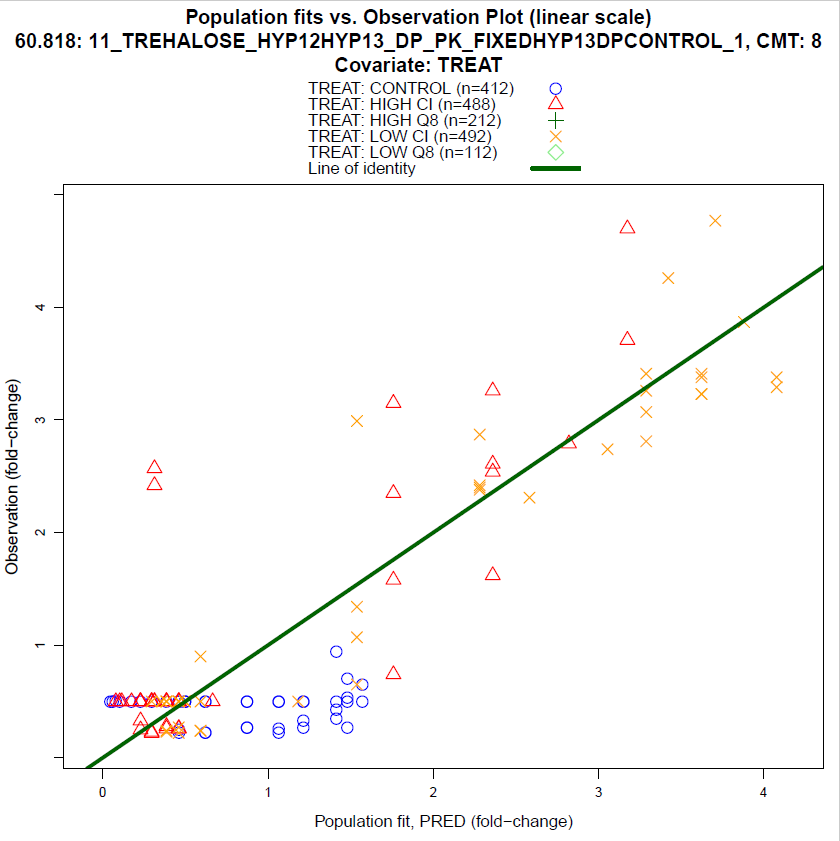  population fit (relative abundance)  Observed data (relative abundance)  r^2^ = 0.76  rBias = 21%  rRMSE= 68% | 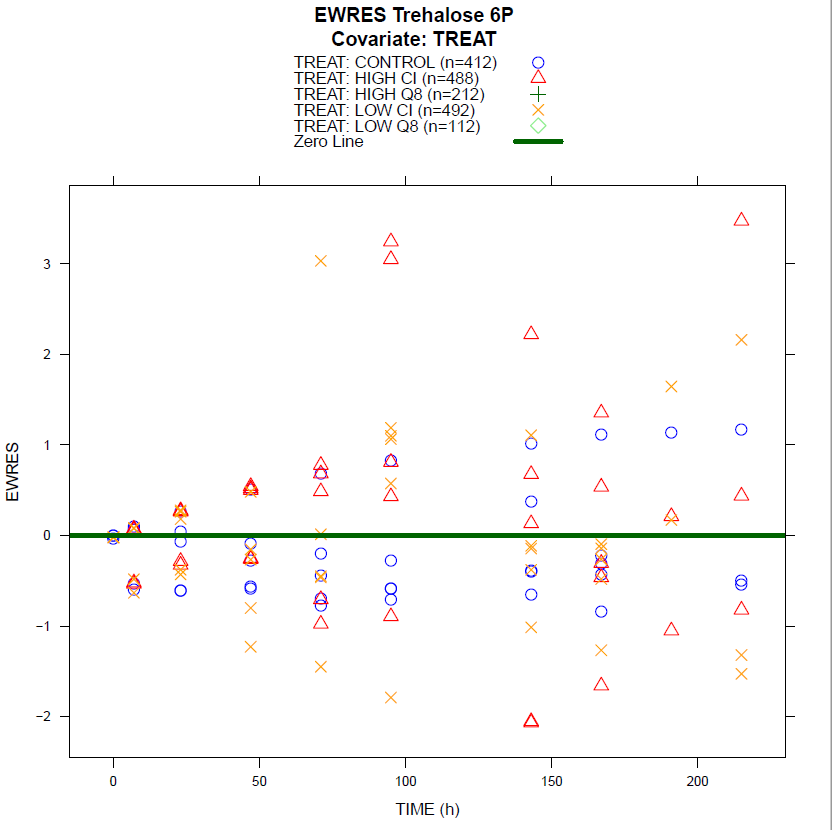 |

**
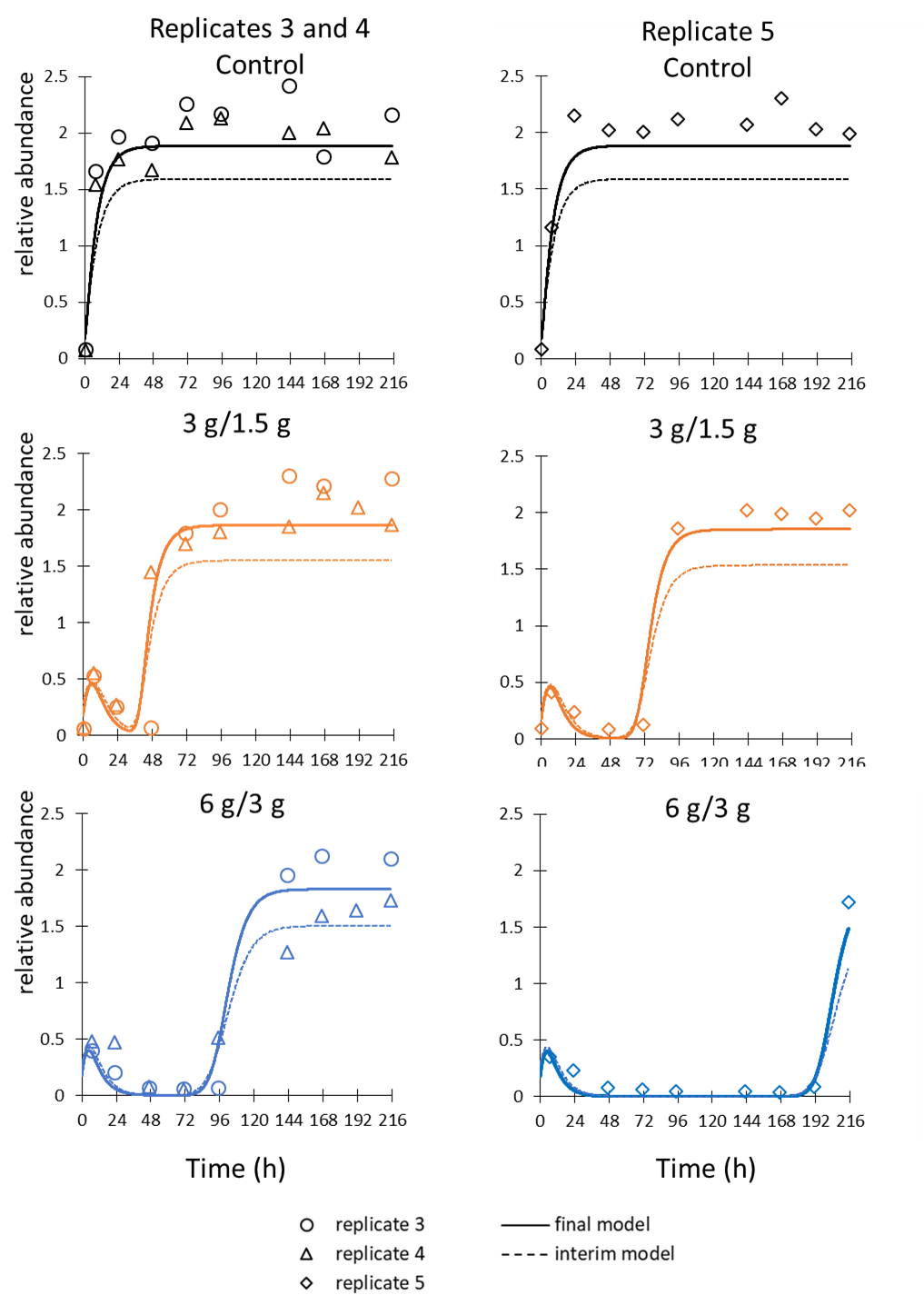
**

**Figure S7** Final model fits (solid lines) and predictions (broken lines) of ornithine after ceftolozane/tazobactam exposure for replicates 3, 4 and 5. Predictions were performed with the metabolite model developed with replicates 1 and 2 and the bacterial model of replicates 3, 4 and 5.

**
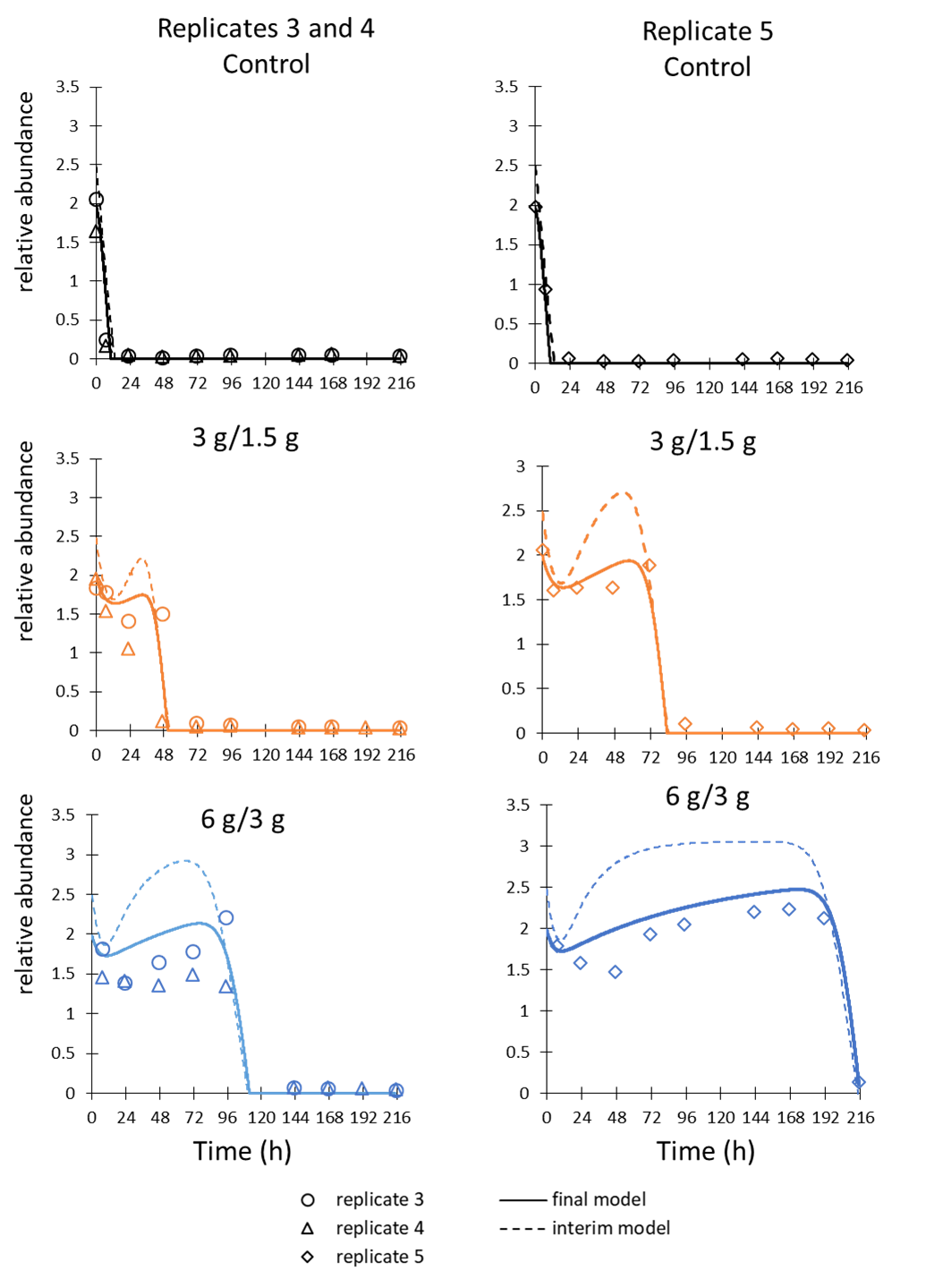
**

**Figure S8** Final model fits (solid lines) and predictions (broken lines) of arginine after ceftolozane/tazobactam exposure for replicates 3, 4 and 5. Predictions were performed with the metabolite model developed with replicates 1 and 2 and the bacterial model of replicates 3, 4 and 5.
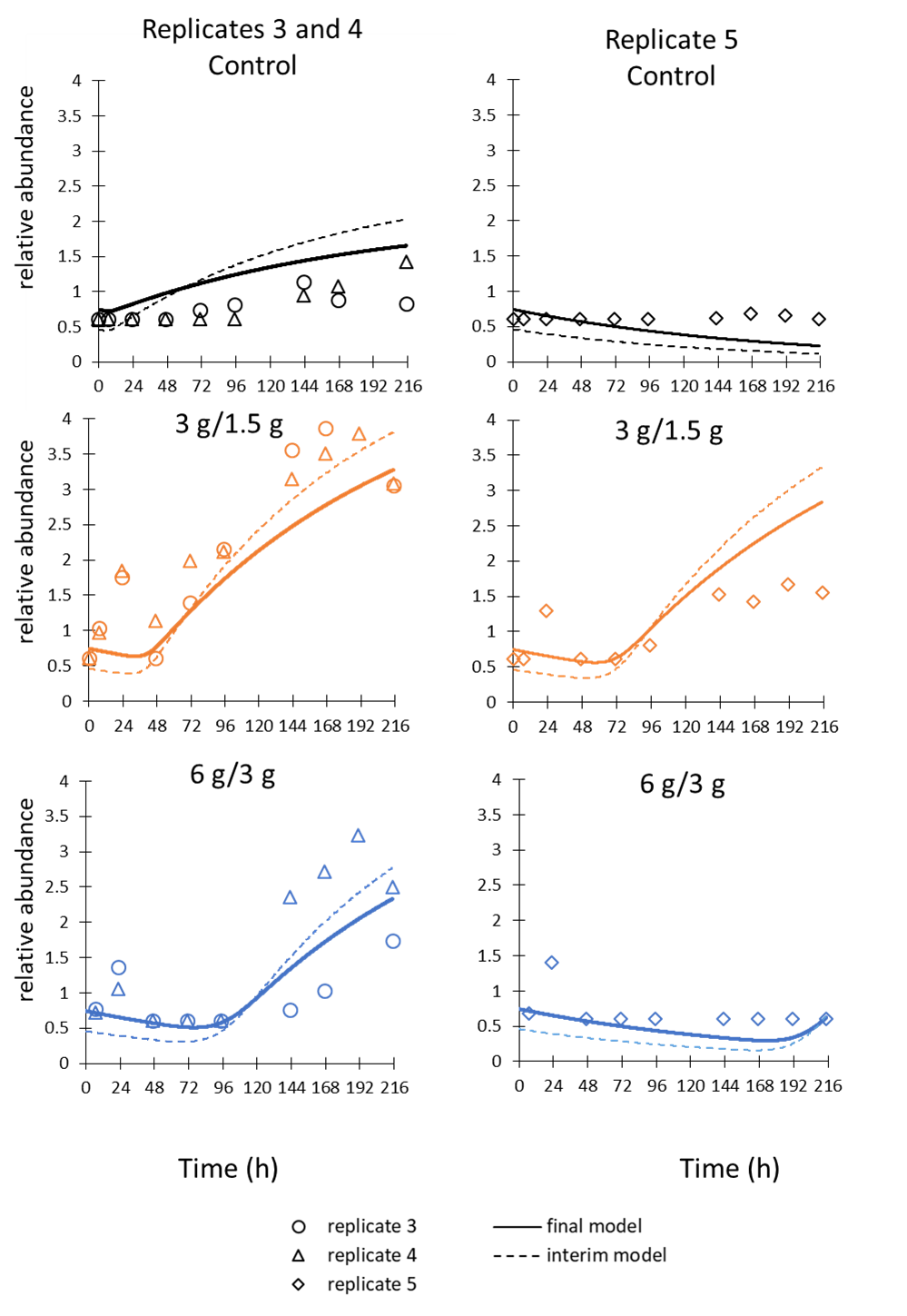


**Figure S9** Final model fits (solid lines) and predictions (broken lines) of ribose 5-phosphate after ceftolozane/tazobactam exposure for replicates 3, 4 and 5. Predictions were performed with the metabolite model developed with replicates 1 and 2 and the bacterial model of 3, 4 and 5.


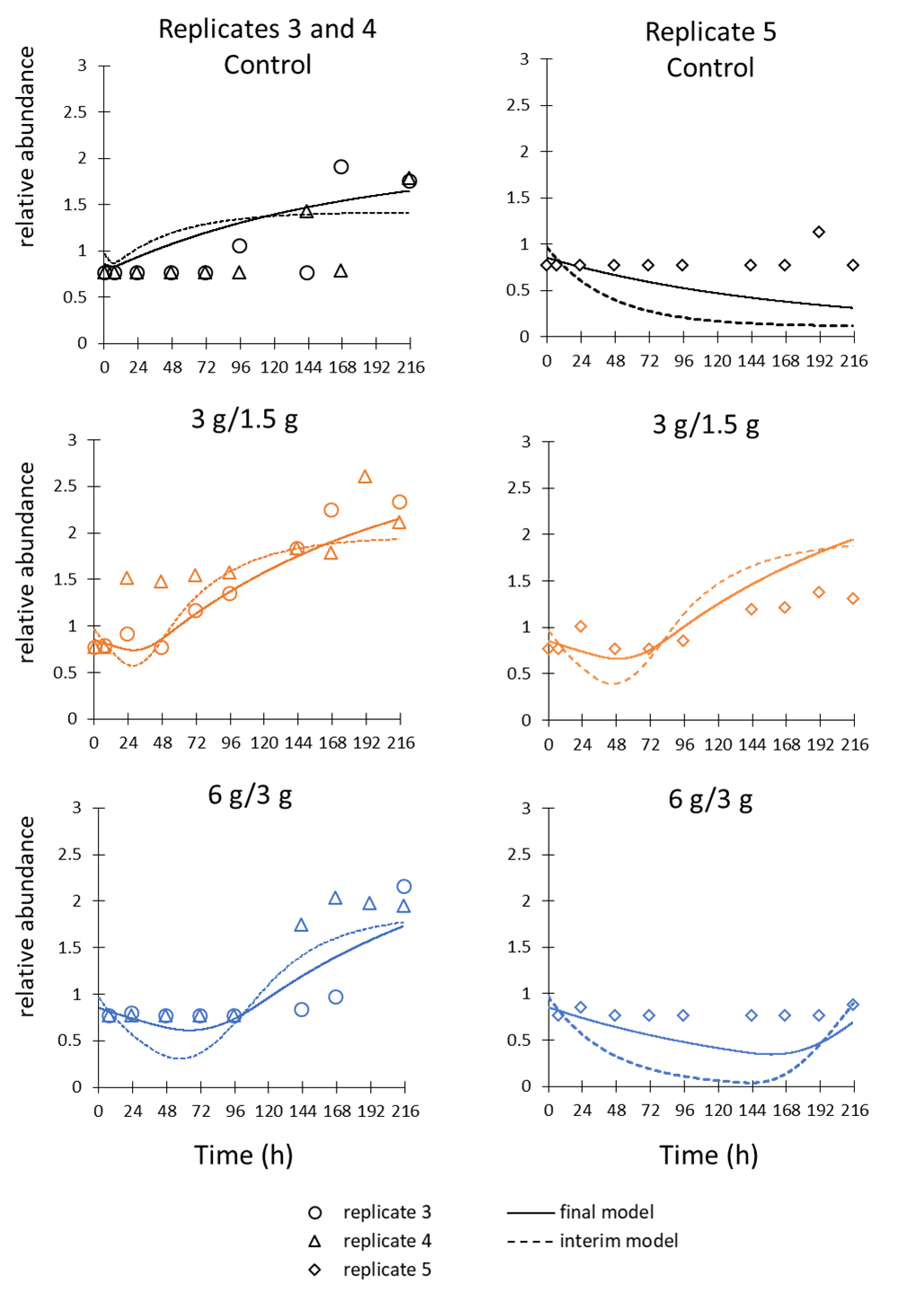


**Figure S10** Final model fits (solid lines) and predictions (broken lines) of sedoheptulose 7-phosphate after ceftolozane/tazobactam exposure for replicates 3, 4 and 5. Predictions were performed with the metabolite model developed with replicates 1 and 2 and the bacterial model of 3, 4 and 5.


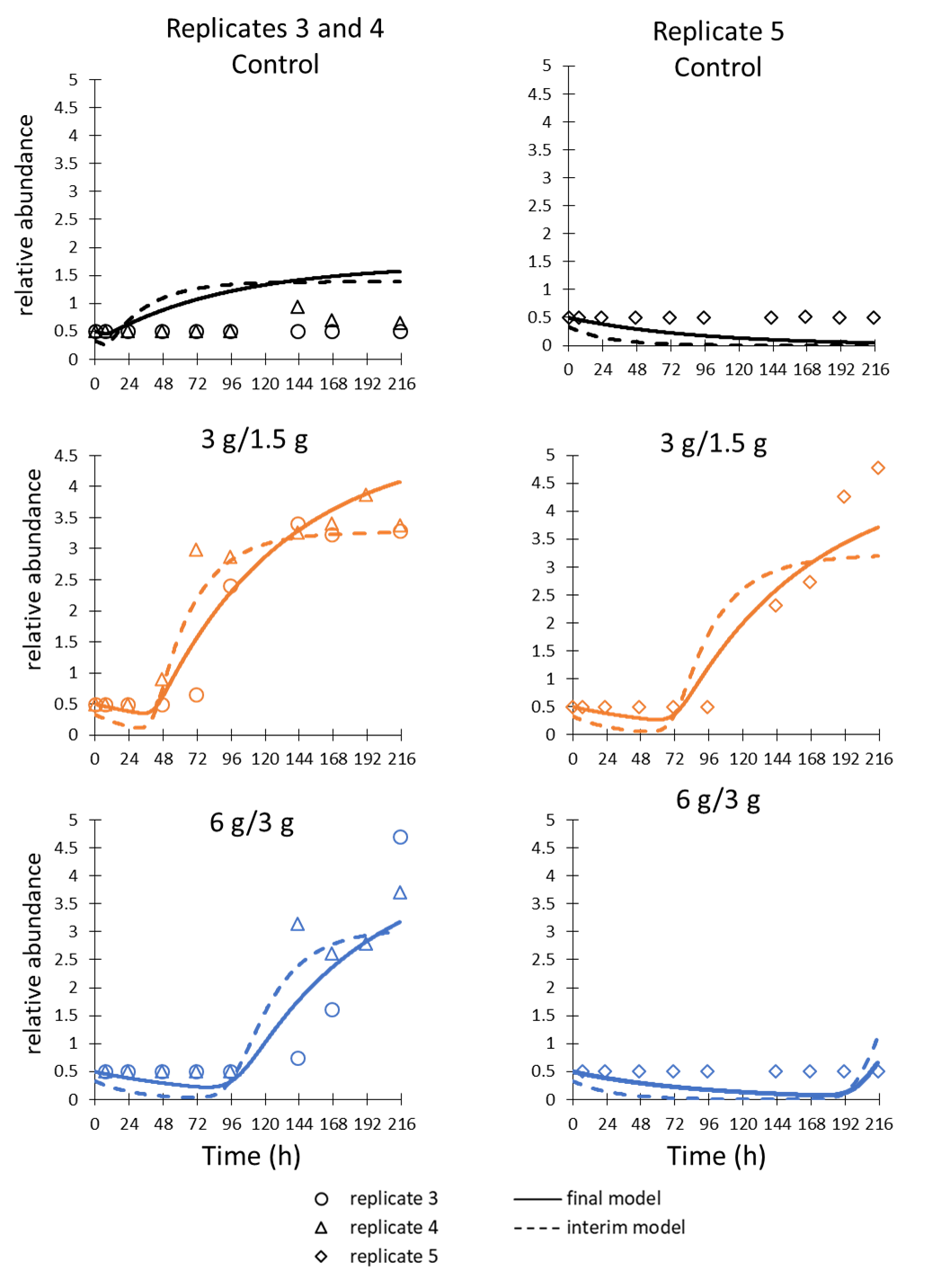


**Figure S11** Final model fits (solid lines) and predictions (broken lines) of trehalose 6-phosphate after ceftolozane/tazobactam exposure for replicates 3, 4 and 5. Predictions were performed with the metabolite model developed with replicates 1 and 2 and the bacterial model of 3, 4 and 5.
